# Identification of Glioblastoma Phosphotyrosine-Containing Proteins with Two-Dimensional Western Blotting and Tandem Mass Spectrometry

**DOI:** 10.1155/2015/134050

**Published:** 2015-05-18

**Authors:** Tianyao Guo, Xiaowei Wang, Maoyu Li, Haiyan Yang, Ling Li, Fang Peng, Xianquan Zhan

**Affiliations:** ^1^Key Laboratory of Cancer Proteomics of Chinese Ministry of Health, Xiangya Hospital, Central South University, 87 Xiangya Road, Changsha, Hunan 410008, China; ^2^Hunan Engineering Laboratory for Structural Biology and Drug Design, Xiangya Hospital, Central South University, 87 Xiangya Road, Changsha, Hunan 410008, China; ^3^State Local Joint Engineering Laboratory for Anticancer Drugs, Xiangya Hospital, Central South University, 87 Xiangya Road, Changsha, Hunan 410008, China; ^4^The State Key Laboratory of Medical Genetics, Central South University, 88 Xiangya Road, Changsha, Hunan 410008, China

## Abstract

To investigate the presence of, and the potential biological roles of, protein tyrosine phosphorylation in the glioblastoma pathogenesis, two-dimensional gel electrophoresis- (2DGE-) based Western blotting coupled with liquid chromatography-electrospray ionization-tandem mass spectrometry (LC-ESI-MS/MS) analysis was used to detect and identify the phosphotyrosine immunoreaction-positive proteins in a glioblastoma tissue. MS/MS and Mascot analyses were used to determine the phosphotyrosine sites of each phosphopeptide. Protein domain and motif analysis and systems pathway analysis were used to determine the protein domains/motifs that contained phosphotyrosine residue and signal pathway networks to clarify the potential biological functions of protein tyrosine phosphorylation. A total of 24 phosphotyrosine-containing proteins were identified. Each phosphotyrosine-containing protein contained at least one tyrosine kinase phosphorylation motif and a certain structural and functional domains. Those phosphotyrosine-containing proteins were involved in the multiple signal pathway systems such as oxidative stress, stress response, and cell migration. Those data show 2DGE-based Western blotting, MS/MS, and bioinformatics are a set of effective approaches to detect and identify glioblastoma tyrosine-phosphorylated proteome and to effectively rationalize the biological roles of tyrosine phosphorylation in the glioblastoma biological systems. It provides novel insights regarding tyrosine phosphorylation and its potential role in the molecular mechanism of a glioblastoma.

## 1. Introduction

Tyrosine phosphorylation that is an addition of phosphogroup (–HPO_3_ to –OH or –H_3_PO_4_ to –NH_2_) to the tyrosine residue is a type of protein posttranslational modification that plays key roles in the signal transduction and participates in many physiological and pathological processes such as growth, proliferation, differentiation, aging, cancer, and inflammatory diseases [1–3]. Tyrosine phosphorylation and dephosphorylation are a reversibly dynamic mechanism that is regulated by protein tyrosine kinases (PTKs) and protein tyrosine phosphatases (PTPs) [4]. Moreover, tyrosine kinase phosphorylation generally occurs within a consensus pattern/motif [R/K]-x(2)-[D/E]-x(3)-Y or [R/K]-x(3)-[D/E]-x(2)-Y (Y = the phosphorylation site) [5–7]. Currently, 518 human protein kinase genes [8] including 90 known tyrosine kinases that include 58 receptor tyrosine kinases (RTKs) [9, 10] and 107 tyrosine phosphatases [11] have been discovered for potential targets of anticancer drugs, most tyrosine kinases are regulated negatively and only activated under certain conditions [8], and interestingly tyrosine kinases accounting for 0.3% of genome contribute to a large proportion (30%) of 100 known dominant oncogenes [10, 12]. Tyrosine phosphorylation (accounting for only ~0.05%) is a low abundance event in the phosphoproteome relative to phosphorylation at the serine (accounting for ~90%) and threonine (accounting for ~10%) residues in eukaryotic cells [1, 3, 10, 13]. However, characterization of altered modification and functional activities of phosphotyrosine-containing proteins in different types of cancers has helped in the discovery of specific tyrosine kinase inhibitors to treat a cancer [9, 14]. Thus, it emphasizes the scientific importance of investigating phosphotyrosine-containing proteins in a cancer.

The most common characteristics of glioblastoma are highly invasive growth and aggressive infiltration into surrounding normal brain, which causes the failure of current therapies to control glioblastoma, with a median survival of 9–12 months in spite of the improvement of the current therapies such as surgery, radiotherapy, and chemotherapy [15]. The molecular mechanisms of glioblastoma remain unclear. It is necessary to discover novel biomarkers for novel therapeutic strategy to control its invasive growth. Many studies have indicated that tyrosine phosphorylation is extensively associated with pathophysiological processes of glioma including angiogenesis [16–21], immune response [22], and invasive growth and migration [23–27]. Tumor angiogenesis is an important reason why glioblastoma is capable of highly invasive growth and aggressive infiltration. Many positive and negative regulating factors of angiogenesis are involved in the tyrosine phosphorylation [16–21], such as vascular endothelial growth factor (VEGF) and its receptor (VEGFR) [16, 17, 21, 28], epidermal growth factor (EGF) and its receptor (EGFR) [15, 19, 20, 29–32], platelet-derived growth factor (PDGF) and its receptor (PDGFR) [29, 33], leucine-rich repeat C4 (LRRC4) [18], the uPA/uPAR system [34], ERK1/2 signaling [35], and the focal adhesion kinase signaling pathway [36, 37]. A series of protein kinases associated with glioma are studied including RTK (EGFR, ErbB2, ErbB3, IGF-IR, and KIT) [30–32, 38–40], Lyn kinase/Src kinase [41], Akt and focal adhesion kinase [27, 36, 37, 42, 43], Janus kianse [44], ABL2/ARG tyrosine kinase [45], ephrin family [46, 47], Fyn related kinase (FRK) [48], STAT-3 [49] and STAT-6 [23], Mer receptor tyrosine kinase [25], and VEGFR-2 tyrosine kinase [28]. The documented literature demonstrates the importance of tyrosine phosphorylation in the pathogenesis of glioma. However, the large-scale detection and identification of phosphotyrosine-containing proteins in glioblastoma are rarely reported. The tyrosine-phosphorylated proteomics analysis is necessary to detect the phosphotyrosine-containing proteins and clarify the potential biological functions of tyrosine phosphorylation in glioblastoma.

MS/MS-identification of phosphotyrosine-containing proteins is hindered by the low abundance of phosphotyrosine-containing proteins [50], and MS-identification of phosphopeptides is also complicated by ion suppression effects because of the high background of nonphosphorylated peptides. Enrichment of phosphotyrosine-containing proteins is essential prior to MS analysis. 2DGE in combination with antiphosphotyrosine antibody is an effective method to relatively enrich and detect phosphotyrosine-containing proteins. In this study, we investigated presence of and the potential biological roles of the tyrosine phosphorylation in a protein in a glioblastoma tissue. Anti-phosphotyrosine antibodies were used to detected phosphotyrosine-containing proteins in a polyvinylidene fluoride (PVDF) membrane that were transferred from a 2D gel with the separated glioblastoma proteins. LC-MS/MS was used to determine the amino acid sequence of those phosphotyrosine-containing proteins that were contained in the immunoreactive-positive 2D gel spots. The protein and phosphotyrosine sites were determined with Mascot software, and the biological functions and pathway networks involved in the modified proteins were achieved with systems pathway analysis. These results provided a platform to investigate phosphotyrosine proteome in human glioblastoma and to explore its potential biological roles of tyrosine phosphorylation in the glioblastoma.

## 2. Materials and Methods

### 2.1. Glioblastoma Tissue

A glioma tissue (male, 57 years old) was obtained from Department of Neurosurgery of Xiangya Hospital, China, and approved by the Xiangya Hospital Medical Ethics Committee of Central South University, China. The glioma tissue was removed from neurosurgery and immediately stored at liquid nitrogen (−196°C). A portion of glioma tissues was used for pathological diagnosis and was diagnosed as grade IV glioblastoma, and the rest was stored in −80°C.

### 2.2. Protein Extraction

A portion of a human glioblastoma tissue (430 mg) was washed with 0.9% NaCl (3 mL, 5×) to remove contaminated blood fully and then was fully grilled in liquid nitrogen. A volume (2 mL) of protein extraction buffer (7 mol/L urea, 2 mol/L thiourea, 40 g/L 3-(3-cholamidopropyl)dimethylammonio-1-propanesulfonate (CHAPS), 100 mmol/L dithiothreitol (DTT), 5 mL/L IPG buffer pH 3–10 NL, and 100 *μ*L of phosphatase inhibitor cocktail (Sigma)) was added and mixed. The mixture was vortexed (2 h) on the ice and centrifuged (1,5000 ×g, 15 min). The supernatant was centrifuged again (1,5000 ×g, 15 min). The supernatant was used as the protein extract and for determination of protein concentration (11.8 *μ*g/*μ*L) with a Bio-Rad 2D Quant kit (Bio-Rad). For an 18 cm immobilized pH gradient (IPG) strip pH 3–10 NL (GE healthcare), a total of 160 *μ*g (13.6 *μ*L) of protein extract were fully mixed with 236.4 *μ*L of protein extraction buffer (7 mol/L urea, 2 mol/L thiourea, 40 g/L CHAPS, 100 mmol/L DTT, 5 mL/L IPG buffer pH 3–10 NL, and a trace of bromphenol blue) and 110 *μ*L of rehydration buffer (7 mol/L urea, 2 mol/L thiourea, 40 g/L CHAPS, 60 mmol/L DTT, 5 mL/L IPG buffer pH 3–10 NL, and a trace of bromophenol blue). The mixture was centrifuged (1,5000 ×g, 15 min). The supernatant was centrifuged again (1,5000 ×g, 15 min). The supernatant is called the “protein sample solution.”

### 2.3. Two-Dimensional Gel Electrophoresis

#### 2.3.1. First Dimension-Isoelectric Focusing (IEF)

The precast IPG strips (pH 3–10 NL; 180 × 3 × 0.5 mm) and 18 cm IPG strip holder were used for IEF on an IPGphor instrument (GH Healthcare) to separate an aliquot (350 *μ*L) of the protein sample solution that contained 160 *μ*g proteins. The IPG strip was rehydrated overnight (~18 h), followed by IEF (20°C) under a running parameter (a gradient at 250 V and 1 h for 125 Vh, a gradient at 1000 V and 1 h for 500 Vh, a gradient at 8,000 V and 1 h for 4,000 Vh, a step and hold at 8,000 V and 4 h for 32,000 Vh, and a step and hold at 500 V and 0.5 h for 250 Vh) to achieve a final 36,875 Vh and ~7.5 h run. After IEF, the IPG strip was processed to the second-dimensional electrophoresis.

#### 2.3.2. Second Dimension-Sodium Dodecyl Sulfate-Polyacrylamide Gel Electrophoresis (SDS-PAGE)

An Ettan DALT II system (Amersham Pharmacia Biotech; analyze up to 12 gels at a time) was used. The 12% PAGE resolving gel (250 × 215 × 1.0 mm) was cast with an Ettan TM DALTsix multigel caster (Amersham BioSciences) that can cast up to 12 gels at a time. The resolving-gel solution for 3 gels was made by mixing 90 mL of 400 g/L acrylamide/bisacrylamide (29 : 1 by weight; cross-linking ratio = 3.3%), 75 mL of 1.5 mol/L tris-HCl pH 8.8, 135 mL of distilled and deionized water, 1.5 mL of 100 g/L ammonia persulfate, and 75 *μ*L of tetramethylethylenediamine (TEMED). The IPG strip with the protein sample was equilibrated in a reducing equilibrium buffer (10 mL; 15 min) that contained 375 mmol/L Trish pH 8.8, 6 mol/L urea, 20 g/L SDS, 200 mol/L glycerol, 20 g/L DTT, and a trace of bromphenol blue. The IPG strip was then equilibrated in an alkylation equilibrium solution (10 mL; 15 min) that contained 25 g/L iodoacetamide instead of 20 g/L DTT. A boiled solution containing 10 g/L low-molecular-weight agarose in the SDS electrophoresis buffer that contained 192 mmol/L glycine, 25 mmol/L Tris, and 1 g/L SDS was used to seal the equilibrated IPG strip to the top of the resolving gel. Second-dimensional electrophoresis was performed in 10 L of tris-glycine-SDS electrophoresis buffer that contained 25 mmol/L tris-base, 192 mmol/L glycine, and 1 g/L SDS with the following conditions: constant 2.5 W/gel for 30 min and then constant 10 W/gel for 340 min.

#### 2.3.3. Silver Staining of Proteins

The 2DGE-separated protein spots were visualized with a modified silver-staining method [51]. The procedure was that (i) the gel was fixed in 250 mL of 50% v/v methanol and 5% v/v acetic acid (20 min), washed in 250 mL of 50% v/v methanol (10 min), and washed in deionized water (10 min); (ii) the gel was sensitized in 250 mL of 0.02% w/v sodium thiosulfate (1 min) and washed with deionized water (1 min, 2 times); (iii) the gel was silver-stained (20 min) in 250 mL of 0.1% w/v silver nitrate plus 200 *μ*L 37% v/v formaldehyde and washed with deionized water (1 min, 2 times); (iv) the gel was developed in 250 mL of 3% w/v sodium carbonate with 100 *μ*L 37% v/v formaldehyde until the desired intensity of staining occurs (usually ca. 3 min); (v) the development was stopped in 250 mL of 5% v/v acetic acid (10 min), and then the gel was washed (5 min) in deionized water and was stored in glycerol (250 mL, 8.8% v/v).

### 2.4. Western Blotting

The proteins separated with 2DGE were transferred to a PVDF membrane (0.8 mA/cm^2^; 1 h, 40 min) with a Pharmacia Biotech Nova Blot semidry transfer instrument. The PVDF membrane with the proteins was blocked (1 h) with a volume (100 mL) of 0.3% bovine serum albumin/tris-buffered saline with 0.1% sodium azide and 0.1% Tween-20 (BSA/TBST). The BSA-blocked PVDF membrane was incubated (5 h, 4°C) with a mouse anti-human phosphotyrosine antibody (Catalogue number MAB3109, Millipore, USA) that was diluted (1 : 1000 = v : v) in a 0.3% BSA/TBST solution. After completion of the incubation with the primary antibody, the membrane was washed with the TBST solution (100 mL; 5 min × 3). The secondary antibody, horse anti-mouse horseradish peroxidase- (HRP-) linked IgG that was purchased from Cell Signaling Technology Inc., USA (Catalogue number 7076), was diluted (1 : 2000 = v : v) in a 0.3% BSA/TBST solution and was added to the blots (1 h, room temperature). The membrane was washed with TBST (100 mL; 10 min × 3), and phosphotyrosine proteins were visualized with ChemiDoc XRS imaging system (Bio Rad, CA, USA). A parallel negative-control experiment was performed to detect any cross-reactivity of the secondary antibody. For the negative-control experiment (the primary antibody was not added), the entire procedure was the same as the Western blotting. The 2DGE gel, after transferring proteins to PVDF membrane, was silver-stained in the same way as described above to detect any remained proteins on the gel for determination of the efficiency of the protein transfer.

### 2.5. Image Analysis of a 2D Gel and of Western Blotting

The scanned images of the silver-stained 2D gels and of the visualized Western blot membranes were input to a PDQuest system (BioRad, version 7.1, Hercules, CA) to generate the synthetic image that contained the Gaussian spots (Gaussian image) with a defined volume (volume = optical density (OD) × width (mm) × length (mm)) and quality [52]. All subsequent spot-matching and analysis steps were performed on the Gaussian spots. In order to minimize the effect of any experimental factor on a spot volume, each spot volume was normalized to the total optical density in each gel image [52].

### 2.6. Determination of Phosphotyrosine-Containing Proteins

The 2D gel spots corresponding to the phosphotyrosine-positive Western blot spot were excised, and the proteins that were contained in 2D gel spots were digested in gel with trypsin [48]. The tryptic peptide mixture was purified with a ZipTipC18 microcolumn (Catalogue number ZTC18S096, Millipore, USA), according to the methods recommended by the manufacturer. For LC-ESI quadrupole time of flight (LC-ESI-qTOF) MS/MS analysis, the purified tryptic peptide mixture was eluted with 6 *μ*L of 850 mL/L acetonitrile plus 1 mL of trifluoroacetic acid (10 cycles) and the elute was air-dried. Before analysis, the dried tryptic peptide mixture was redissolved in 6 *μ*L of 50 mL/L acetonitrile plus 1 mL/L formic acid. The purified peptide mixture was subjected to LC-ESI-qTOF MS/MS analysis. Briefly, the tryptic peptides from 2D gel spots were loaded onto a C18 precolumn for concentrations and fast desalting and then eluted to the reversed-phase column for separation. MS/MS spectra were performed in data-depended mode in which up to four precursor ions above an intensity threshold of 7 counts/seconds (cps) were selected for MS/MS analysis from each survey scan. The obtained MS/MS data were used for protein database searching.

For MS/MS database searching, the peptide sequence tag format file that was generated from MS/MS data with MassLynx version 4.0 software was input into the Mascot search engine to search protein against the Swiss-Prot database (release date December 1, 2013; 541954 sequences; 192668437 residues; Homosapiens 20274 sequences). A mass tolerance of 0.3 Da for both parent (MS) and fragmented (MS/MS) ions, allowance for up to one trypsin miscleavage, fixed amino acid modification consisting of cysteine carbamidomethylation, variable amino acid modifications consisting of methionine oxidation, and tyrosine phosphorylation were used. MS/MS ion score threshold was determined to produce a false-positive rate less than 5% for a significant hit (*P* < 0.05). The false-positive rate was calculated with 2 ∗ reverse/(reverse + forward)/100. In the current study, the least MS/MS ion score threshold was 35 and a false-positive rate was approximately 3.1%. Each protein was determined with MS/MS-based amino acid sequences. If protein was identified with only one peptide, its MS/MS spectrum was further checked manually. Each phosphotyrosine-containing peptide was checked manually. Each manual check must consider those factors: high-quality MS/MS spectrum with good signal-to-noise ratio, matched main ion peaks, a good b- or y-ion series, a high intensity of the corresponding precursor ion, the corresponding good LC peaks, and so forth. Also, a blank gel on the margin on a 2D gel was analyzed in parallel to remove any contaminated proteins including trypsin and keratin from the statistically significant results based the MS/MS protein database searching.

Because tyrosine phosphorylation commonly occurs within tyrosine kinase phosphorylation motif, each MS/MS-derived protein sequence was input into the ScanProsite program (http://prosite.expasy.org/scanprosite) to determine its protein domains and tyrosine kinase phosphorylation motifs. For the protein without an MS/MS-characterized phosphotyrosine site, it must contain a tyrosine kinase phosphorylation motif to be determined as a phosphotyrosine immune-positive protein.

### 2.7. Bioinformatics Analysis

Gene-ontology (GO) analysis was used to get more insight on the biological significance of phosphotyrosine-containing proteins with exploring the relationship between the biological terms and associated genes using the NIH-DAVID software (version 6.7, http://david.abcc.ncifcrf.gov/summary.jsp). GO terms with computed *P* value of less than 0.05 were considered as significantly enriched terms. Homosapiens were selected to limit annotations. Three structured ontologies were chosen to allow the description of biological process, molecular function, and cellular component. Phosphotyrosine-containing proteins were divided into different clusters according to biological function. The proteins within a cluster were close from a biological perspective and correspondingly far from the proteins in other clusters. Moreover, the Swiss-Prot accession numbers of phosphotyrosine-containing proteins were saved as a text file that was input into Cytoscape version 3.0.2 (http://www.cytoscape.org), BiNGO plugin 2.44 downloaded from Cytoscape manage plugin was used to analyze the enriched biological processes and molecular functions, and CytoKegg plugin was used to mine the signaling pathway networks that involved the phosphotyrosine-containing proteins.

Ingenuity pathway analysis (IPA) was used to obtain further insight into potential cellular pathways that might be modified as a result of protein changes identified in this present study. IPA automatically generated networks of gene, protein, small molecule, drug, and disease associations on the basis of “hand-curated” data held in a proprietary database. The identifiers (Swiss-Prot identification number) of phosphotyrosine-containing proteins were uploaded as an Excel spreadsheet file into the Ingenuity software (Ingenuity Systems, Redwood City, CA, USA). Each human identification number was mapped to its corresponding molecule in the ingenuity pathway knowledge base. The statistically significant signaling pathway networks, canonical pathways, biofunctions, and toxfunctions were generated to involve those phosphotyrosine-containing proteins and address the effects of protein tyrosine phosphorylation on those biological pathway systems. Each network, pathway, biofunction, and toxfunction was presented as a graph that indicated the molecular relationship between proteins.

## 3. Results and Discussion

### 3.1. DGE-Based Western Blot Detection of Phosphotyrosine-Containing Proteins

**Figure 1:**
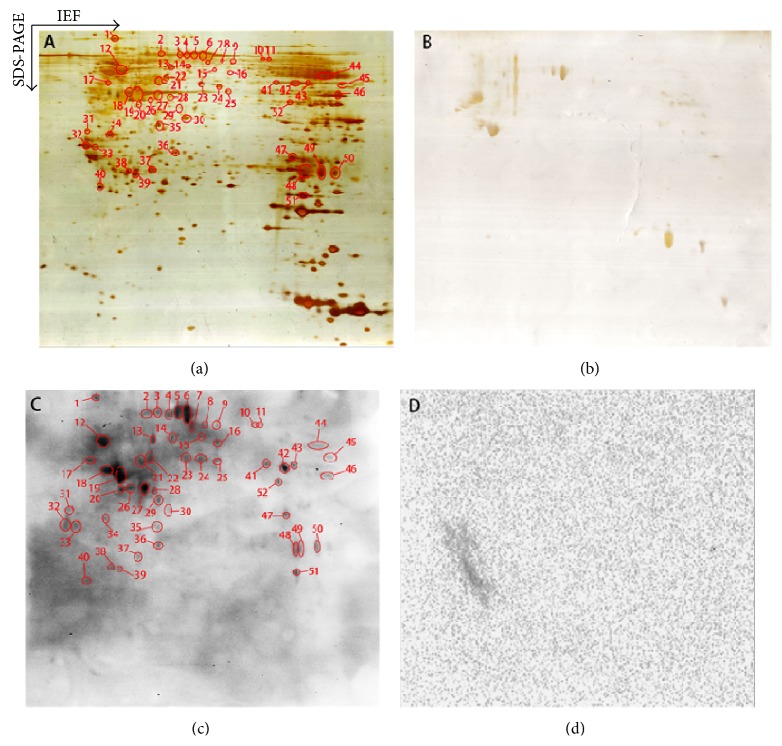
Two-dimensional gel electrophoresis-based Western blot analysis of antiphosphotyrosine proteins in a glioblastoma tissue (160 *μ*g protein per 2D gel). (a) Silver-stained image on a 2D gel before transfer of proteins to a PVDF membrane. (b) Silver-stained image on a 2D gel after transfer of proteins to a PVDF membrane. (c) Western blotting image of antiphosphotyrosine proteins (antiphosphotyrosine antibodies + secondary antibody). (d) Negative control of Western blotting to show the cross-reaction of the secondary antibody (only the secondary antibody, no antiphosphotyrosine antibody).

*Ca.* 900 protein spots were detected in each silver-stained 2D gel. Most protein spots were distributed within a region of pI 4–8 and *M*_*r*_ 15–100 kDa. Those phosphotyrosine immunopositive proteins that were transferred onto a PVDF membrane were detected with an anti-human phosphotyrosine antibody (Figure 1). Moreover, a parallel negative-control experiment was carried out to determine any cross-reactivity of secondary antibody.  Figure 1(a) shows the silver-stained 2D gel image before proteins were transferred onto a PVDF membrane.  Figure 1(b) shows the corresponding silver-stained 2D gel image after proteins were transferred onto a PVDF membrane and demonstrates that at least 92% proteins [(900 − 70)/900] were transferred onto the PVDF membrane.  Figure 1(c) shows the Western blot image with the labeled positive phosphotyrosine-immunoreactivity, 51 phosphotyrosine immunopositive Western blot spots were detected, and the corresponding silver-stained protein spots were labeled in Figure 1(a).  Figure 1(d) shows there was no cross-reactivity of secondary antibody to further confirm the positive Western blot spots in Figure 1(c).

### 3.2. LC-ESI-MS/MS Characterization of Phosphotyrosine-Containing Proteins

The proteins that were contained in each 2D gel spot corresponding to the positive phosphotyrosine immunoreactivity were excised and subjected to in-gel digestion with trypsin and purification of tryptic peptides, followed by LC-ESI-MS/MS analysis. The protein and phosphotyrosine site were determined with MS/MS data. Those proteins without MS/MS-characterized phosphotyrosine site were subjected to the ScanProsite analysis to determine their tyrosine kinase phosphotyrosine motifs. In order to consolidate the protein with a phosphotyrosine-immunoreactivity, at least one tyrosine kinase phosphotyrosine motif was contained in that protein amino acid sequence. A total of 36 proteins were identified with MS/MS from 51 phosphotyrosine immunopositive spots (Tables 1 and 2 and Supplemental Table 1  in Supplementary Material available online at http://dx.doi.org/10.1155/2015/134050). In order to consolidate the identification of phosphotyrosine-containing proteins, 12 proteins without predicted Tyr-phosphomotif and without MS/MS-characterized phosphotyrosine sites (Supplemental Table 1) were considered as uncertain phosphotyrosine-containing proteins. Thus, a total of 24 phosphotyrosine-containing proteins were identified in a glioblastoma tissue (Tables 1 and 2). Of them, 15 positive phosphotyrosine-immunoreactivity proteins were identified and summarized in Table 1, and 9 phosphoproteins with MS/MS-characterized phosphotyrosine sites were identified and summarized in Table 2.

Table 1 contained the spot number, Swiss-Prot access number, protein name, molecular weight, pI, Mascot score, the number of matched unique peptides, and tyrosine kinase phosphorylation motifs; those phosphotyrosine-containing proteins were heat shock protein 90 alpha, heat shock protein 90 beta, heat shock 70 kDa protein 1A/1B, tubulin alpha-1A chain, tubulin alpha-1B chain, tubulin alpha-8 chain, cytoplasmic actin 1, glial fibrillary acidic protein, beta-actin-like protein 2, L-lactate dehydrogenase B chain, 14-3-3 protein epsilon, annexin A5, apolipoprotein A-I, and alpha-enolase. Table 2 contained the spot number, Swiss-Prot access number, protein name, phosphotyrosine-containing peptide sequence, peptide mass, Mascot ion score, and tyrosine kinase phosphorylation motifs; those phosphotyrosine-containing proteins were receptor-type tyrosine-protein phosphatase S, Arf-GAP with Rho-GAP domain, ANK repeat and PH domain-containing protein 1, centrosomal protein of 192 kDa, plexin-D1, HEAT repeat-containing protein 5B, zinc finger protein 569, beta-hexosaminidase subunit alpha, homeobox protein Hox-A1, and pre-mRNA-processing-splicing factor 8.

### 3.3. Protein Domains/Motifs-Based Functional Recognition of Phosphotyrosine-Containing Proteins

**Figure 2:**
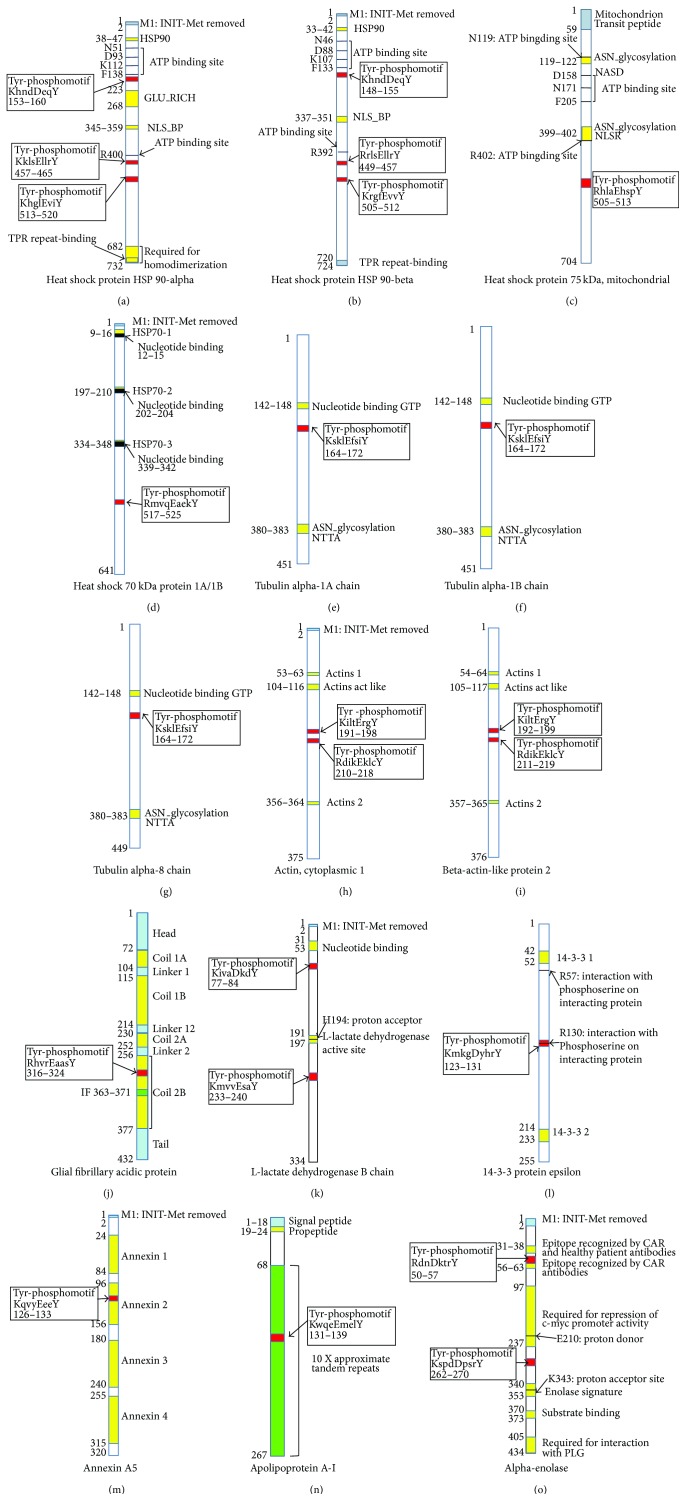
Tyrosine kinase phosphorylation motif and functional domains of putative phosphotyrosine-containing proteins in a glioblastoma tissue. INIT-Met, initiator methionine; HSP90, heat shock protein 90 family signature; GLU_RICH, glutamic acid-rich region profile; NLS_BP, bipartite nuclear localization signal profile; TPR, tetratricopeptide; ASN, N-glycosylation site; HSP70, heat shock protein 70 family signature; GTP, guanosine triphosphate; PLG, plasminogen.

**Figure 3:**
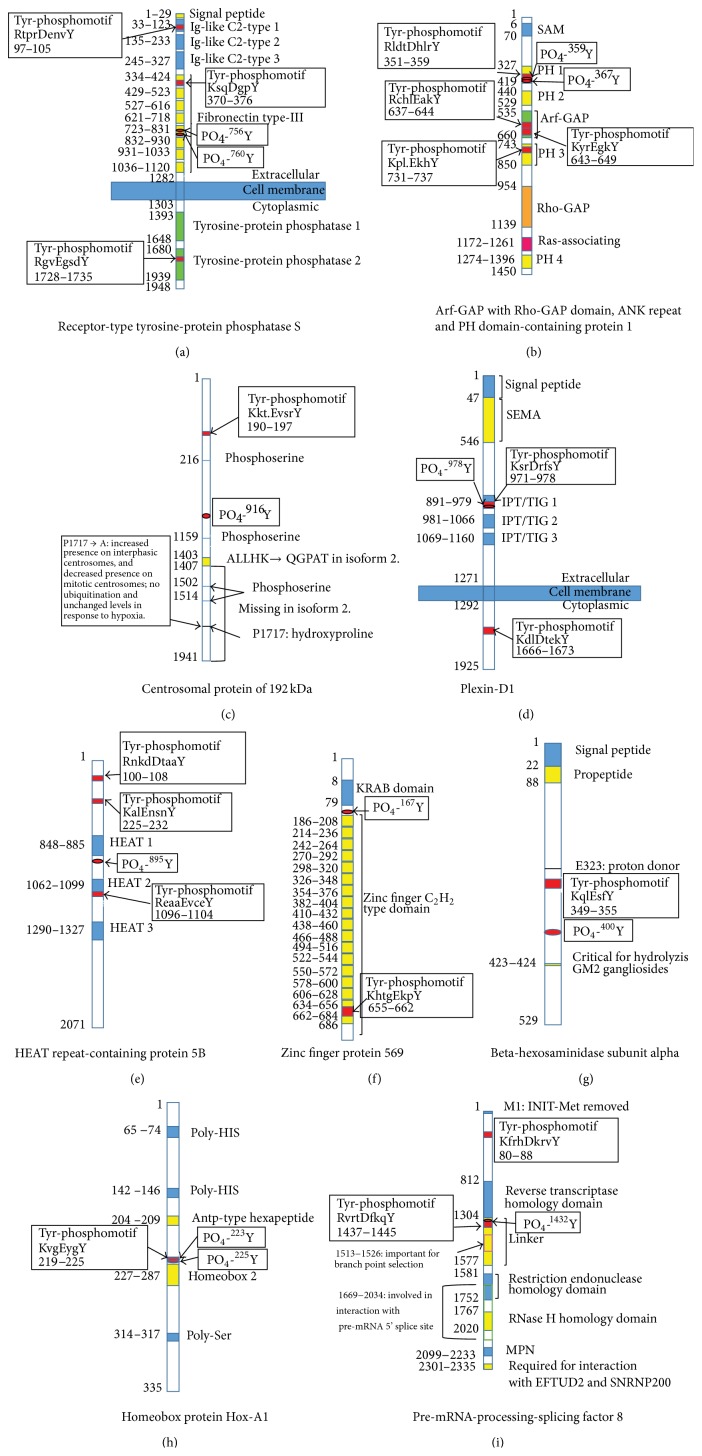
Phosphotyrosine sites, tyrosine kinase phosphorylation motifs, and functional domains of phosphotyrosine-containing proteins in a glioblastoma tissue. SAM, the sterile *α* motif; PH, pleckstrin homology; Arf-GAP, ADP ribosylation factor GTPase-activating protein domain; Rho-GAP, Rho GTPase-activating proteins domain; SEMA, semaphorins; IPT/TIG, Ig-like, plexins, transcription factors/trigger factor-like protein; KRAB, Krueppel-associated box; GM2, the second monosialic ganglioside; HIS, histidine; MPN, domain at Mpr1p and Pad1p N-termini; EFTUD2, elongation factor Tu GTP-binding domain-containing protein 2; SNRNP200, small nuclear ribonucleoprotein 200 kDa.

Each protein contained certain structural and functional domains or motifs. Identification of those domains and motifs is helpful to understand the structure and functions of each individual protein. Moreover, tyrosine phosphorylation commonly occurs within a characteristic Tyr-phosphomotif. The identification of Tyr-phosphomotifs further consolidated each identified phosphotyrosine-containing protein. The protein domains and motifs were determined with literature-based bioinformatics and ScanProsite analyses. Each protein contained at least one tyrosine kinase phosphorylation motif (Figures 2 and 3; Tables 1 and 2). It further confirmed the results of 2D-Western blot antiphosphotyrosine immunity reaction. Figures 2 and 3 illustrate all the functional domains of each phosphoprotein.

Figures 2(a) and 2(b) show the functional domains and motifs of heat shock protein 90- (HSP90-) alpha and (HSP90-) beta, which contains 3 Try-phosphomotifs, 5 ATP-binding sites, 1 NLS_BP motif, and 1 TPR repeat-binding. HSP90-alpha and HSP90-beta are molecular chaperones promoting the maturation, structural maintenance, and proper regulation of specific target proteins that are involved in cell cycle control and signal transduction and undergo a functional cycle linked to its ATPase activity [53–57]. HSP90-alpha is a homodimer, interacts with STUB1 and UBE2N, and is involved in the ubiquitination systems. HSP90-beta is also a homodimer and interacts with p53/TP53. They are involved in stress response. Mitochondrial HSP75 (Figure 2(c)) contains a Tyr-phosphomotif, 3 ATP binding sites, and two glycosylation motifs; it is a chaperone expressing an ATPase activity and involved in maintaining mitochondrial function and polarization; it interacts with tumor necrosis factor type 1 receptor; and as a negative regulator of mitochondrial respiration, it modulates the balance between oxidative phosphorylation and aerobic glycolysis [58–60]. HSP70 1A/1B (Figure 2(d)) contains 3 nucleotide binding sites and 1 Tyr-phosphomotif and is involved in stress-induced damage. Tubulin alpha-1A, tubulin alpha-1B, and tubulin alpha-8 chains (Figures 2(e), 2(f), and 2(g)) contain a nucleotide binding GTP site, ASN_glycosylation, and 1 Tyr-phosphomotif. Tubulin alpha is the major constituent of microtubules and forms dimmer with beta chains, which binds two moles of GTP, one at an exchangeable site on the beta chain and one at a nonexchangeable site on the alpha chain [61, 62]. Cytoplasmic 1 actin (Figure 2(h)) and beta-actin-like protein 2 (Figure 2(i)) contain the same 2 ACTIN domains, 1 ACTIN ACT LIKE domains, and 2 Tyr-phosphomotifs. Actins are highly conserved proteins that are involved in various types of cell motility and are ubiquitously expressed in all eukaryotic cells. Its phosphorylation would affect cell motility [63]. Glial fibrillary acidic protein (Figure 2(j)) contains 1 Tyr-phosphomotif and 3 coil domain, is a class-III intermediate filament, and is a cell-specific marker that distinguishes astrocytes from other glial cells during the development of the central nervous system [64]. L-lactate dehydrogenase B chain (Figure 2(k)) contains 2 Tyr-phosphomotifs, 1 nucleotide binding site, and 1 L-lactate dehydrogenase active site; it is homotetramer in cytoplasm and catalyzes lactate to produce pyruvate and NADH [65]. 14-3-3 protein epsilon (Figure 2(l)) contains two 14-3-3 domains, two recognitions of phosphoserine motifs, and one Tyr-phosphomotif; it is homodimer in cytoplasm and participates in the regulation of a wide-range of signaling pathways [66]. Annexin A5 (Figure 2(m)) contains 1 Tyr-phosphomotif and 4 ANNEXIN domains that bind calcium and phospholipid acts, and it acts as an indirect inhibitor of the thromboplastin-specific complex [67]. Apolipoprotein A-I (Figure 2(n)) contains 10 approximate tandem repeats and 1 Tyr-phosphomotif. It is a secreted protein and is involved in the reverse transport of cholesterol from tissues to the liver for excretion by promoting cholesterol efflux from tissues and by acting as a cofactor for the lecithin cholesterol acyltransferase and participates in lipid metabolism [68]. Alpha-enolase (Figure 2(o)) contains 2 Tyr-phosphomotifs and 1 enolase signature and 1 substrate binding region. Alpha-enolase is a multifunctional enzyme that is involved in various processes such as growth control, hypoxia tolerance, and allergic responses, also functions in the intravascular and pericellular fibrinolytic system [69], and has been used as diagnostic marker for many tumors [70].

Figure 3(a) shows the protein domains and motifs of receptor-type tyrosine-protein phosphatase S, including 3 Ig-like C2-type domains, 1 fibronectin type-III domain, 1 transmembrane region, 2 tyrosine-protein phosphatases, and 3 Tyr-phosphomotifs; it is involved in receptor desensitization, signal transduction, and membrane localization [71]. Arf-GAP with Rho-GAP domain, ANK repeat, and PH domain-containing protein 1 (Figure 3(b)) contains 4 PH domains, 1 Ras-associating domain, 1 Rho-GAP domain, 1 Arf-GAP domain, and 4 Tyr-phosphomotifs; it is a phosphatidylinositol 3,4,5-trisphosphate-dependent GTPase-activating protein that modulates actin cytoskeleton remodeling by regulating ARF and RHO family members [72]. Centrosomal protein of 192 kDa (Figure 3(c)) contains 3 phosphoserine sites and 1 Tyr-phosphomotif; its hydroxylation promotes ubiquitination [73]. Plexin-D1 (Figure 3(d)) is a transmembrane protein, containing 1 SEMA domain, 3 IPT/TIG domains, and 2 Tyr-phosphomotifs; it plays an important role in cell-cell signaling and in regulating the migration of a wide spectrum of cell types [74]. HEAT repeat-containing protein 5B (Figure 3(e)) contains 3 HEAT domains and 3 Tyr-phosphomotifs and is involved in the regulation of cell cycle [75]. Zinc finger protein 569 (Figure 3(f)) contains 19 zinc finger C_2_H_2_ type domains, 1 KRAB domain, and 1 Tyr-phosphomotif; it involved transcription regulation and suppresses MAPK signaling pathway [76]. Beta-hexosaminidase subunit alpha (Figure 3(g)) contains a critical motif for hydrolysis GM2 gangliosides and a propeptide and 1 Tyr-phosphomotif; it is responsible for the degradation of GM2 gangliosides and a variety of other molecules containing terminal N-acetyl hexosamines, in the brain and other tissues [77]. Homeobox protein Hox-A1 (Figure 3(h)) contains 2 Poly-HIS, 1 homeobox 2, 1 poly-Ser, Antp-type hexapeptide, and 1 Tyr-phosphomotif; it is involved in transcription regulations [78]. Pre-mRNA-processing-splicing factor 8 (Figure 3(i)) contains a reverse transcriptase homology domain, a restriction endonuclease homology domain, an RNase H homology domain, an MPN, and 2 Tyr-phosphomotifs; it is involved in mRNA processing and functions as a scaffold that mediates the ordered assembly of spliceosomal proteins and snRNAs [79].

### 3.4. Systems Biology Strategy-Based Recognition of Biological Functions of Phosphotyrosine-Containing Proteins

Functional enrichment analysis was performed for 24 phosphotyrosine-containing proteins identified from a glioblastoma tissue; their biological functions were rationalized in glioblastoma. All the 24 phosphotyrosine-containing proteins were accepted for GO analysis and CytoScape BINGO analysis and were hierarchically classified into 4 clusters (Table 3). Proteins within the same cluster were coregulated proteins and might have similar biological functions in the glioblastoma. Those phosphoproteins were involved in multiple biological functions altered in glioblastoma, including oxidative stress and stress response and cell migration. Significantly, GO analysis showed that different biological functions changed during the pathophysiological processes of glioblastoma.

**Figure 4:**
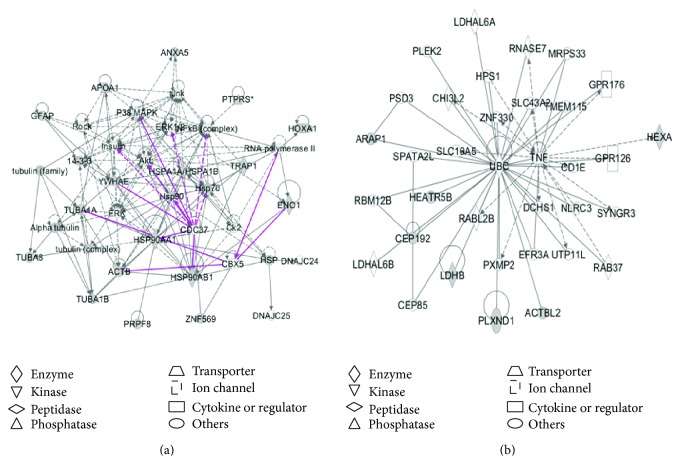
Significant signaling pathway networks mined from phosphotyrosine-containing proteins in a glioblastoma tissue. Significant signaling pathway networks that are involved in human glioblastoma phosphotyrosine-containing proteins and that function in (a) cancer, organismal injury and abnormalities, reproductive system disease, and developmental disorder (merged Networks 1 and 3 in the Supplemental Table 3) and (b) cell morphology, cellular assembly and organization, cellular function, and maintenance (Network 2). A black solid edge denotes a direct relationship between two nodes (molecules: proteins; genes). A black unsolid edge denotes an indirect relationship between two nodes (molecules: proteins; genes). The various shapes of nodes denote the different functions. A curved line means intracellular translocation; a curved arrow means extracellular translocation.

Pathway network analysis further revealed the potential biological functions of those characterized phosphotyrosine-containing proteins in a human glioblastoma. Among 24 phosphotyrosine-containing proteins (Supplemental Table 2), all those 24 phosphotyrosine-containing proteins were accepted for IPA analysis to determine significant pathway networks, canonical pathways, and disease biological events. Two statistically significant pathway networks were identified to involve the phosphotyrosine-containing proteins (Figure 4 and Supplemental Table 3). Those nodes in Figure 4 correspond to those molecules (genes; proteins) that were summarized in Supplemental Table 3. Network A (Figure 4(a)) functions in cancer, organismal injury and abnormalities, reproductive system disease, and developmental disorder (merged from Networks 1 and 3 in the Supplemental Table 3) and includes 39 nodes (genes; proteins). Among those 39 nodes, 17 phosphotyrosine-containing proteins (44% of the total nodes) were identified with MS. ERK, Akt, P38MAPK, Jnk, HSP90, HSP70, tubulin complex, NF-*κ*B complex, and insulin play key roles in this network. Network B (Figure 4(b)) functions in cell morphology, cellular assembly and organization, cellular function, and maintenance (corresponded to Network 2 in the Supplemental Table 3) and includes 35 nodes (genes; proteins). Among those 35 nodes, 7 phosphotyrosine-containing proteins (20% of the total nodes) were identified with MS. TNF, UBC, and CEP192 play key roles in this network.

**Figure 5:**
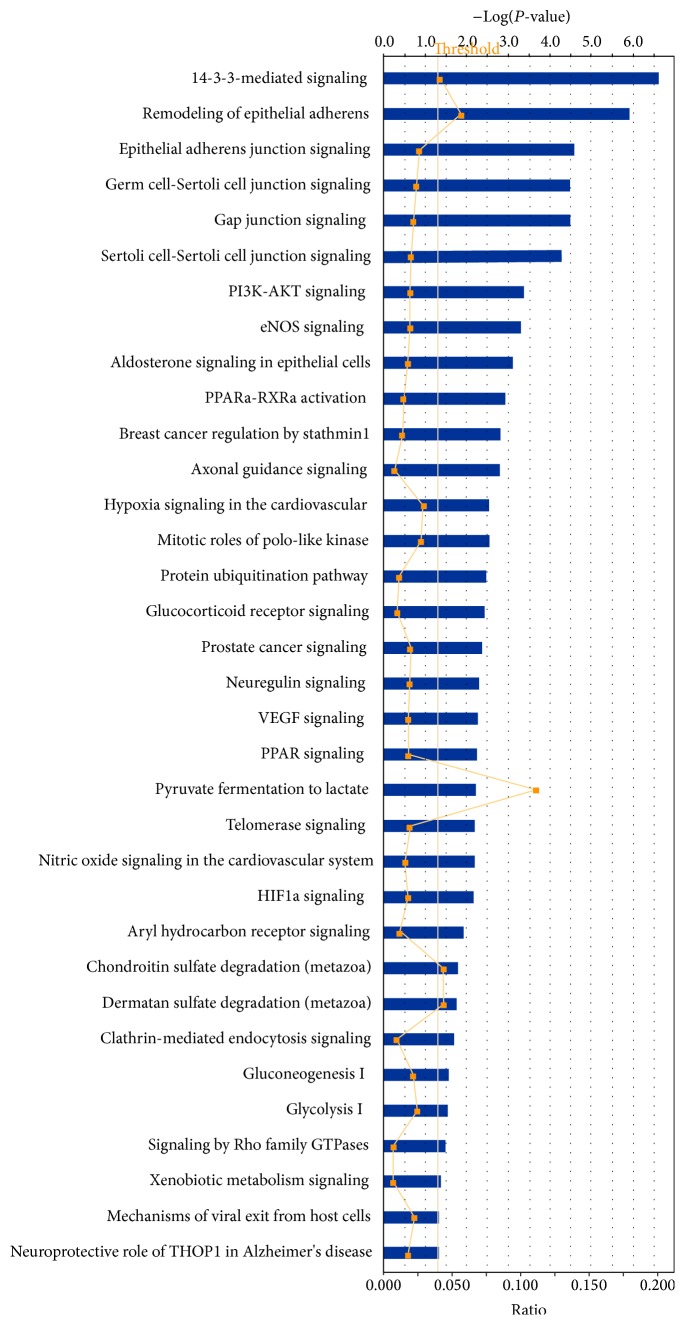
Significant canonical pathways that are involved with phosphotyrosine-containing proteins in a glioblastoma tissue. Each significant canonical pathway was collected as in Supplemental Figure 1.

**Figure 6:**
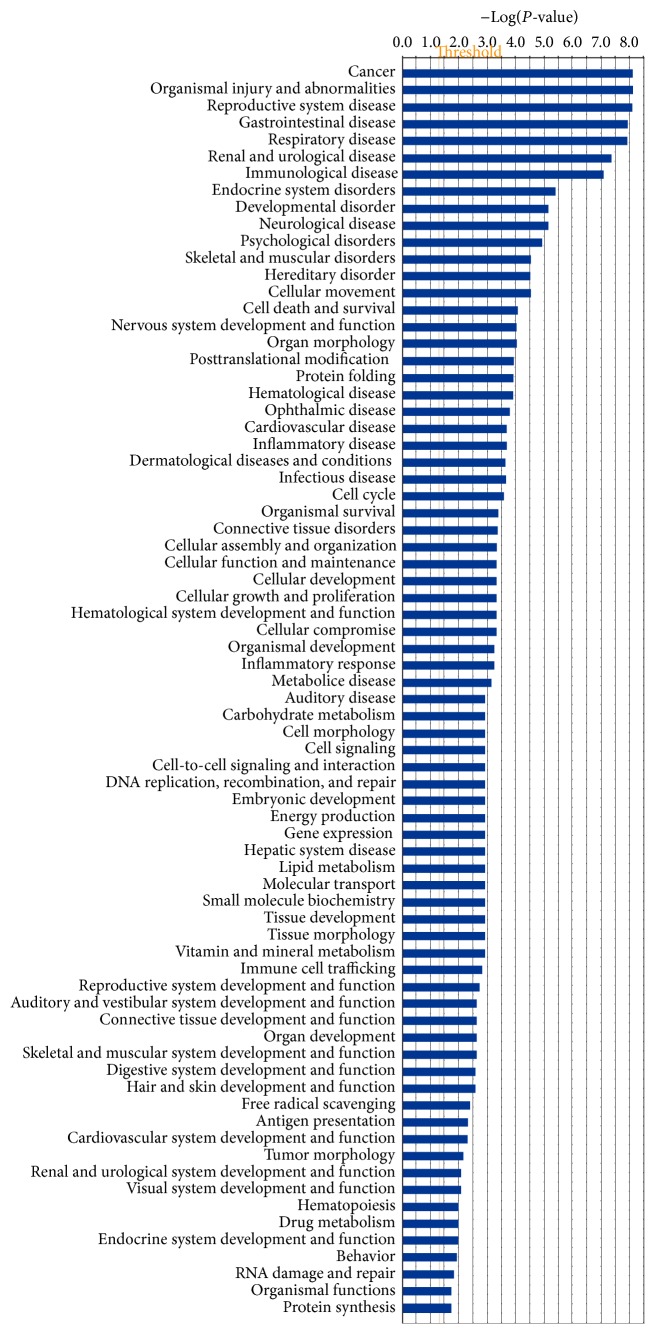
Significant disease biological events that are involved with phosphotyrosine-containing proteins in a glioblastoma tissue.

Among those sets of glioblastoma phosphotyrosine-containing protein data, 36 statistically significant canonical pathways were identified to involve those phosphotyrosine-containing proteins (Figure 5). Each detailed statistically significant canonical pathway was collected in Supplemental Figure 1, including 14-3-3-mediated signaling, cell cycle G2/M DNA damage checkpoint regulation, eNOS signaling, gap junction signaling, gluconeogenesis I, glycolysis I, HIF1a signaling, PI3K-AKT signaling, protein ubiquitination pathway, pyruvate fermentation to lactate, signaling by Rho family GTPases, and VEGF signaling. Moreover, 74 statistically significant disease biological events (Figure 6) involved those phosphotyrosine-containing proteins, including cancer, endocrine system disorders, neurological disease, inflammatory disease, cell cycle dysregulation, energy metabolism, immunity, and protein synthesis. Those pathway networks, canonical pathways, and disease biological events provided a functional profile of those phosphotyrosine-containing proteins in human glioblastoma.

**Figure 7:**
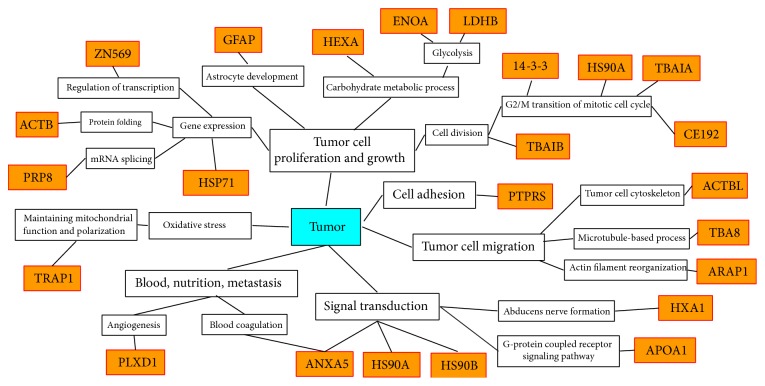
Experimental data-based diagram that rationalizes phosphotyrosine-containing proteins in the glioma biological system. The orange frame means identified phosphotyrosine-containing proteins. ANXA5, annexin A5; PLXD1, plexin-D1; TRAP1, TNFR-associated protein 1 (heat shock protein 75 kDa, mitochondrial); PRP8, pre-mRNA-processing-splicing factor 8; ACTB, actin, cytoplasmic 1; ZN569, zinc finger protein 569; GFAP, glial fibrillary acidic protein; HEXA, beta-hexosaminidase subunit alpha; ENOA, alpha-enolase; LDHB, L-lactate dehydrogenase B chain; 14-3-3, 14-3-3 protein; HSP90A, heat shock protein HSP 90-alpha; TBAIA, tubulin alpha-1A chain; CE192, centrosomal protein of 192 kDa; ACTBL, beta-actin-like protein 2; TBA8, tubulin alpha-8 chain; ARAP1, Arf-GAP with Rho-GAP domain, ANK repeat, and PH domain-containing protein 1; HXA1, homeobox protein Hox-A1; APOA1, apolipoprotein A-I; and HSP90B, heat shock protein HSP 90-beta.

Furthermore, extensive literature-based analysis proposed an experimental data-based diagram that rationalizes the identified phosphotyrosine-containing proteins in the glioma biological system (Figure 7). Those phosphotyrosine-containing proteins are involved in tumor cell proliferation, growth, adhesion, migration, angiogenesis, tumor metastasis, blood supply, nutrition, signal transduction, and oxidative stress to associate the processes of tumor pathogenesis.

## 4. Conclusions

The present study provides new insights to explore the presence and biological significance of tyrosine phosphorylation in the pathological processes of glioblastoma. The combination of Western blotting and LC-ESI-MS/MS is an effective method to detect and characterize phosphotyrosine-containing proteins in human glioblastoma proteome. 2DGE-based Western blotting can preseparate and enrich proteins with a similar pI and *M*_*r*_. LC can real-time preseparate and enrich those tryptic peptides before mass spectrometry analysis. MS/MS can accurately locate each phosphotyrosine site. Protein domain/motif analysis can locate the phosphotyrosine site within the corresponding protein domains. Each identified phosphotyrosine-containing protein contains at least one Tyr-phosphomotif. Pathway analysis-based bioinformatics can reveal the signaling pathway networks that involve phosphoproteins. This methodology provides a basis to comprehensively investigate the phosphotyrosine-containing proteome in the human glioblastoma, especially to achieve our goal to detect and characterize glioma-related phosphotyrosine-containing proteins in a program to clarify the basic molecular mechanisms of glioblastoma formation. Further investigation is needed to determine the biological consequences of the identified tyrosine phosphorylation events and their relevance to the pathogenic mechanisms of glioblastoma.

## Supplementary Material

Supplemental Table 1: Proteins without tyrosine phosphorylated motif, identified with 2DGE-based Western blotting and MS/MS
Supplemental Table 2: Phosphotyrosine-containing proteins identified in a human glioblastoma tissue for IPA analysis
Supplemental Table 3: Signaling pathway networks that involve phosphotyrosine-containing proteins identified from human glioblastoma
Supplemental Figure 1: Significant canonical pathways involved phosphotyrosine-containing proteins in a glioblastoma tissue

## Figures and Tables

**Table 1:** Putative phosphotyrosine-containing proteins identified with 2DGE-based Western blotting and tandem mass spectrometry.

Spot number	Swiss-Prot number	Protein name	Molecular weight	pI	Mascot score	Number of matched unique peptides	Predicted Tyr-phosphomotif	Tyr-phosphomotif position
1	P07900	Heat shock protein HSP 90-alpha	85006	4.94	258	5	KhndDeqY KklsEllrY KhglEviY	153–160 457–465 513–520
1	P08238	Heat shock protein HSP 90-beta	83554	4.97	237	5	KhndDeqY RrlsEllrY KrgfEvvY	148–155 449–457 505–512
1	Q12931	Heat shock protein 75 kDa, mitochondrial	80345	8.3	138	1	RhlaEhspY	505–513
2	P08107	Heat shock 70 kDa protein 1A/1B	70294	5.48	52	2	RmvqEaekY	517–525
12	Q71U36	Tubulin alpha-1A chain	50788	4.94	1591	12	KsklEfsiY	164–172
12	P68363	Tubulin alpha-1B chain	50804	4.94	1521	12	KsklEfsiY	164–172
12	Q9NY65	Tubulin alpha-8 chain	50746	4.94	689	7	KsklEfaiY	164–172
18, 27	P60709	Actin, cytoplasmic 1	42052	5.29	138	2	KiltErgY RdikEklcY	191–198 210–218
27	Q562R1	Beta-actin-like protein 2	42318	5.39	105	2	KiltErgY RdvkEklcY	192–199 211–219
21, 38, 47, 48, 51	P14136	Glial fibrillary acidic protein	49907	5.42	1331	8	RhvrEaasY	316–324
30	P07195	L-lactate dehydrogenase B chain	36900	5.71	130	4	KivaDkdY KmvvEsaY	77–84 233–240
32, 33	P62258	14-3-3 protein epsilon	29326	4.63	223	4	KmkgDyhrY	123–131
34	P08758	Annexin A5	35971	4.94	95	2	KqvyEeeY	126–133
38, 39	P02647	Apolipoprotein A-I	30759	5.56	219	5	KwqeEmelY	131–139
42, 43	P06733	Alpha-enolase	47139	7.01	387	5	Rdn.DktrY KspdDpsrY	50–57 262–270

**Table 2:** Phosphotyrosine-containing proteins identified with 2DGE-based Western blotting and tandem mass spectrometry.

Spot number	Swiss-Prot number	Protein name	Monoisotopic mass of neutral peptide *M*_*r*_ (calc)	Phosphotyrosine-containing peptide sequences	Predicted Tyr-phosphomotif	Tyr-phosphomotif position
3	Q13332	Receptor-type tyrosine-protein phosphatase S	1940.8	GY^*^QVHY^*^VRMEGAEAR	RtprDenvY Ksq.Dgp.Y Rgv.EgsdY	97–105 370–376 1728–1735
42	Q13332	Receptor-type tyrosine-protein phosphatase S	1940.8	GY^*^QVHY^*^VRMEGAEAR	RtprDenvY Ksq.Dgp.Y Rgv.EgsdY	97–105 370–376 1728–1735
6	Q96P48	Arf-GAP with Rho-GAP domain, ANK repeat and PH domain-containing protein 1	1496.5	(RLDTDHLR)Y^*^FDSNKDAY^*^SK	RldtDhlrY RchlEak.Y Kyr.Egk.Y Kpl.Ekh.Y	351–359 637–644 643–649 731–737
6	Q8TEP8	Centrosomal protein of 192 kDa	1792.8	KDVLDFGDLTY^*^GGWK	Kkt.EvsrY	190–197
12	Q9Y4D7	Plexin-D1	2110.0	(KSRDR)FSY^*^VLPLVHSLEPTM^#^GPK	Ksr.DrfsY Kdl.DtekY	971–978 1666–1673
21	Q9P2D3	HEAT repeat-containing protein 5B	1325.6	M^#^AQY^*^SFDKLK	RnkdDtaaY Kal.EnsnY ReaaEvceY	100–108 225–232 1096–1104
21	Q5MCW4	Zinc finger protein 569	1511.6	KEHCEY^*^NEPVK	KhtgEkp.Y	655–662
42	P06865	Beta-hexosaminidase subunit alpha	1187.5	EDIPVNY^*^MK	Kql.Esf.Y	349–355
46	P49639	Homeobox protein Hox-A1	1967.9	TGKVGEY^*^GY^*^LGQPNAVR	Kvg.Eyg.Y	219–225
47	Q6P2Q9	Pre-mRNA-processing-splicing factor 8	1325.6	HTLAY^*^DKGWR	KfrhDkrvY RvrtDfkqY	80–88 1437–1445

Y^*^ = phosphotyrosine residue, M^#^ = oxidated methine residue.

**Table 3:** The functional categories of phosphotyrosine-containing proteins identified by GO analysis.

Category	Term	Count	*P* Value	Phosphotyrosine-containing proteins
Annotation cluster 1				
INTERPRO	Heat shock protein Hsp90, conserved site	3	1.66*E* − 05	HS90A, HS90B, TRAP1
SP_PIR_KEYWORDS	Stress response	4	6.51*E* − 05	HS90A, HSP71, HS90B, TRAP1
PIR_SUPERFAMILY	Heat shock protein, HSP90/HTPG types	3	1.04*E* − 04	HS90A, HS90B, TRAP1
INTERPRO	Heat shock protein Hsp90	3	1.09*E* − 04	HS90A, HS90B, TRAP1
SP_PIR_KEYWORDS	Nucleotide-binding	9	5.06*E* − 04	HS90A, HSP71, TBA1A, HS90B, TBA1B, ACTB, TBA8, ACTBL, TRAP1
SMART	HATPase_c	3	6.82*E* − 04	HS90A, HS90B, TRAP1
GOTERM_MF_FAT	Unfolded protein binding	4	6.91*E* − 04	HS90A, HSP71, HS90B, TRAP1
INTERPRO	ATP-binding region, ATPase-like	3	7.08*E* − 04	HS90A, HS90B, TRAP1
SP_PIR_KEYWORDS	Chaperone	4	8.54*E* − 04	HS90A, HSP71, HS90B, TRAP1
GOTERM_MF_FAT	Nucleotide binding	10	0.003658702	HS90A, HSP71, TBA1A, HS90B, TBA1B, ACTB, LDHB, TBA8, ACTBL, TRAP1
GOTERM_MF_FAT	Purine ribonucleotide binding	9	0.004063562	HS90A, HSP71, TBA1A, HS90B, TBA1B, ACTB, TBA8, ACTBL, TRAP1
GOTERM_MF_FAT	Ribonucleotide binding	9	0.004063562	HS90A, HSP71, TBA1A, HS90B, TBA1B, ACTB, TBA8, ACTBL, TRAP1
GOTERM_BP_FAT	Response to unfolded protein	3	0.004383705	HS90A, HSP71, HS90B
GOTERM_MF_FAT	Purine nucleotide binding	9	0.005349089	HS90A, HSP71, TBA1A, HS90B, TBA1B, ACTB, TBA8, ACTBL, TRAP1
GOTERM_BP_FAT	Response to protein stimulus	3	0.009707935	HS90A, HSP71, HS90B
KEGG_PATHWAY	Antigen processing and presentation	3	0.018271588	HS90A, HSP71, HS90B
SP_PIR_KEYWORDS	ATP-binding	6	0.018307877	HS90A, HSP71, HS90B, ACTB, ACTBL, TRAP1
GOTERM_BP_FAT	Protein folding	3	0.025155073	HS90A, HS90B, TRAP1
Annotation Cluster 2				
INTERPRO	Alpha tubulin	3	5.96*E* − 05	TBA1A, TBA1B, TBA8
KEGG_PATHWAY	Pathogenic *Escherichia coli* infection	4	3.53*E* − 04	TBA1A, TBA1B, ACTB, TBA8
INTERPRO	Tubulin/FtsZ, 2-layer sandwich domain	3	3.78*E* − 04	TBA1A, TBA1B, TBA8
INTERPRO	Tubulin, conserved site	3	4.14*E* − 04	TBA1A, TBA1B, TBA8
INTERPRO	Tubulin	3	4.51*E* − 04	TBA1A, TBA1B, TBA8
INTERPRO	Tubulin/FtsZ, GTPase domain	3	4.51*E* − 04	TBA1A, TBA1B, TBA8
PIR_SUPERFAMILY	Tubulin	3	0.001118145	TBA1A, TBA1B, TBA8
GOTERM_BP_FAT	Cellular protein complex assembly	4	0.001418705	HS90A, TBA1A, TBA1B, TBA8
GOTERM_BP_FAT	Macromolecular complex assembly	6	0.001848247	HS90A, TBA1A, TBA1B, ANXA5, TBA8, APOA1
GOTERM_BP_FAT	Protein polymerization	3	0.002113041	TBA1A, TBA1B, TBA8
GOTERM_BP_FAT	Macromolecular complex subunit organization	6	0.002466419	HS90A, TBA1A, TBA1B, ANXA5, TBA8, APOA1
GOTERM_BP_FAT	Protein complex assembly	5	0.004757666	HS90A, TBA1A, TBA1B, ANXA5, TBA8
GOTERM_BP_FAT	Protein complex biogenesis	5	0.004757666	HS90A, TBA1A, TBA1B, ANXA5, TBA8
GOTERM_MF_FAT	Structural constituent of cytoskeleton	3	0.005698133	GFAP, TBA1B, ACTB
GOTERM_BP_FAT	Cellular macromolecular complex assembly	4	0.009429911	HS90A, TBA1A, TBA1B, TBA8
GOTERM_BP_FAT	Microtubule-based movement	3	0.010778579	TBA1A, TBA1B, TBA8
GOTERM_CC_FAT	Cytoskeleton	7	0.012358854	CE192, TBA1A, GFAP, TBA1B, ACTB, TBA8, ACTBL
GOTERM_BP_FAT	Cellular macromolecular complex subunit organization	4	0.012902263	HS90A, TBA1A, TBA1B, TBA8
GOTERM_MF_FAT	Structural molecule activity	5	0.014607667	TBA1A, GFAP, TBA1B, ACTB, TBA8
KEGG_PATHWAY	Gap junction	3	0.02084525	TBA1A, TBA1B, TBA8
SP_PIR_KEYWORDS	Microtubule	3	0.030784033	TBA1A, TBA1B, TBA8
GOTERM_MF_FAT	GTPase activity	3	0.041221688	TBA1A, TBA1B, TBA8
GOTERM_CC_FAT	Microtubule cytoskeleton	4	0.045760129	CE192, TBA1A, TBA1B, TBA8
GOTERM_CC_FAT	Cytoskeletal part	5	0.048220584	CE192, TBA1A, GFAP, TBA1B, TBA8
GOTERM_BP_FAT	Microtubule-based process	3	0.048340219	TBA1A, TBA1B, TBA8
UP_SEQ_FEATURE	Nucleotide phosphate-binding region:GTP	3	0.048546013	TBA1A, TBA1B, TBA8
Annotation Cluster 3				
GOTERM_CC_FAT	Melanosome	3	0.007590334	HS90A, HS90B, 1433E
GOTERM_CC_FAT	Pigment granule	3	0.007590334	HS90A, HS90B, 1433E
GOTERM_MF_FAT	Protein domain specific binding	4	0.013570111	HS90A, TBA1A, HS90B, 1433E
GOTERM_CC_FAT	Cytoplasmic membrane-bounded vesicle	4	0.045968066	HS90A, HS90B, 1433E, APOA1
GOTERM_CC_FAT	Membrane-bounded vesicle	4	0.049793741	HS90A, HS90B, 1433E, APOA1
Annotation Cluster 4				
SP_PIR_KEYWORDS	Disease mutation	7	0.009381239	HEXA, TBA1A, GFAP, ACTB, PRP8, LDHB, APOA1

## Abbreviations

BSA: Bovine serum albumin
CHAPS: 3-(3-Cholamidopropyl)dimethylammonio-1-propanesulfonate
DTT: Dithiothreitol
EGF: Epidermal growth factor
EGFR: Epidermal growth factor receptor
FRK: Fyn related kinase
ESI: Electrospray ionization
GO: Geneontology
HSP: Heat shock protein
IEF: Isoelectric focusing
IPA: Ingenuity pathway analysis
IPG: Immobilized pH gradient
LC: liquid chromatography
LRRC4: Leucine-rich repeat C4
MS: Mass spectrometry
MS/MS: Tandem mass spectrometry
OD: Optical density
PDGF: Platelet-derived growth factor
PDGFR: Platelet-derived growth factor receptor
PTK: Protein tyrosine kinase
PTP: Protein tyrosine phosphatase
PVDF: Polyvinylidene fluoride
qTOF: Quadrupole time of flight
RTK: Receptor tyrosine kinase
SDS: Sodium dodecyl sulfonate
SDS-PAGE: Sodium dodecyl sulfate-polyacrylamide gel electrophoresis
2D: Two-dimensional
2DGE: Two-dimensional gel electrophoresis
TBS: Tris-buffered saline
TEMED: Tetramethylethylenediamine
VEGF: Vascular endothelial growth factor
VEGFR: Vascular endothelial growth factor receptor.
