# Supplementary material for: Identification of Glioblastoma Phosphotyrosine-Containing Proteins with Two-Dimensional Western Blotting and Tandem Mass Spectrometry
Source: Biomed Res Int. 2015 May 18;2015:134050. doi: 10.1155/2015/134050 (PMC4450212; doi:10.1155/2015/134050)

Supplemental Table 1. Proteins without tyrosine phosphorylated motif, identified with 2DGE-based Western blotting and MS/MS

Spot number	Swiss-Prot No.	Protein name	Molecular weight	pI	Mascot score	Number of matched unique peptides	Predicted Tyr-Phospho-motif
3, 4, 6, 13, 23	P02768	Serum albumin	71317	5.92	1321	15	No
12	P08670	Vimentin	53676	5.06	725	7	No
12	Q13885	Tubulin beta-2A chain	50274	4.78	119	3	No
12	Q13509	Tubulin beta-3 chain	50856	4.83	119	3	No
12	Q9H853	Putative tubulin-like protein alpha-4B	27819	7.71	45	1	No
27, 33	P12277	Creatine kinase B-type	42902	5.34	730	7	No
37	P09936	Ubiquitin carboxyl-terminal hydrolase isozyme L1	25151	5.33	76	3	No
40	P13693	Translationally-controlled tumor protein	19697	4.84	44	1	No
44, 47	A8MPP1	Putative ATP-dependent RNA helicase DDX11-like protein 8	102944	7.28	47	1	No
47	P00915	Carbonic anhydrase 1	28909	6.59	113	2	No
48, 49, 50	P01834	Ig kappa chain C region	11773	5.58	163	3	No
51	P04179	Superoxide dismutase [Mn], mitochondrial	24878	8.35	136	2	No

Supplemental Table 2. Phosphotyrosine-containing proteins identified in a human glioblastoma tissue for IPA analysis.

ID	Gene name	Protein name	Subcellular location	Function	Drugs
P07900	HSP90AA1	Heat shock protein 90kDa alpha (cytosolic), class A member 1	Cytoplasm	Enzyme	Alvespimycin, retaspimycin, luminespib, cisplatin
P08238	HSP90AB1	Heat shock protein 90kDa alpha (cytosolic), class B member 1	Cytoplasm	Enzyme	Alvespimycin, retaspimycin, cisplatin
Q12931	TRAP1	TNF receptor-associated protein 1	Cytoplasm	Enzyme	
HSPA1A	HSPA1A/HSPA1B	Heat shock 70kDa protein 1A	Cytoplasm	Other	
Q71U36	TUBA1A	Tubulin, alpha 1a	Cytoplasm	Other	Epothilone B, colchicine/probenecid, larotaxel, eribulin, davunetide, vintafolide, milataxel, cevipabulin, gemcitabine/paclitaxel, docetaxel/prednisone, cyclophosphamide/prednisone/vincristine, docetaxel/hydrocortisone, cyclophosphamide/docetaxel, gemcitabine/vinorelbine, cyclophosphamide/daunorubicin/imatinib/prednisone/vincristine, cyclophosphamide/topotecan/vincristine, docetaxel/gemcitabine, docetaxel/gemcitabine/vincristine, irinotecan/vincristine, irinotecan/temozolomide/vincristine,

					bevacizumab/paclitaxel, cyclophosphamide/docetaxel/epirubicin/5-fluorouracil/trastuzumab, docetaxel/trastuzumab, trastuzumab/vinorelbine, gemcitabine/oxaliplatin/paclitaxel, cyclophosphamide/epirubicin/vincristine, docetaxel/irinotecan, docetaxel/5-fluorouracil/oxaliplatin, capecitabine/docetaxel/gemcitabine, L-asparaginase/prednisone/vincristine, cyclophosphamide/etoposide/prednisone/rituximab/vincristine, cyclophosphamide/vinorelbine, cyclophosphamide/mitoxantrone/prednisone/vincristine, cyclophosphamide/etoposide/prednisone/vincristine, cyclophosphamide/prednisone/rituximab/vincristine, cyclophosphamide/mitoxantrone/prednisone/rituximab/vincristine, plinabulin, docetaxel, albendazole, mebendazole, vinflunine, vinorelbine, vincristine, vinblastine, paclitaxel, podophyllotoxin, colchicine
P68363	TUBA1B	Tubulin, alpha 1b	Cytoplasm	Other	
Q9NY65	TUBA8	Tubulin, alpha 8	Cytoplasm	Other	Epothilone B, colchicine/probenecid, larotaxel, eribulin, davunetide, vintafolide, milataxel, cevipabulin, gemcitabine/paclitaxel, docetaxel/prednisone, cyclophosphamide/prednisone/vincristine,

	docetaxel/hydrocortisone, cyclophosphamide/docetaxel, gemcitabine/vinorelbine, cyclophosphamide/daunorubicin/imatinib/prednisone/vincristine, cyclophosphamide/topotecan/vincristine, docetaxel/gemcitabine, docetaxel/gemcitabine/vincristine, irinotecan/vincristine, irinotecan/temozolomide/vincristine, bevacizumab/paclitaxel, cyclophosphamide/docetaxel/epirubicin/5-fluorouracil/trastuzumab, docetaxel/trastuzumab, trastuzumab/vinorelbine, gemcitabine/oxaliplatin/paclitaxel, cyclophosphamide/epirubicin/vincristine, docetaxel/irinotecan, docetaxel/5-fluorouracil/oxaliplatin, capecitabine/docetaxel/gemcitabine, L-asparaginase/prednisone/vincristine, cyclophosphamide/etoposide/prednisone/rituximab/vincristine, cyclophosphamide/vinorelbine, cyclophosphamide/mitoxantrone/prednisone/vincristine, cyclophosphamide/etoposide/prednisone/vincristine, cyclophosphamide/prednisone/rituximab/vincristine, cyclophosphamide/mitoxantrone/prednisone/rituxima

					b/vincristine, plinabulin, docetaxel, vinflunine, vinorelbine, vincristine, vinblastine, paclitaxel, podophyllotoxin, colchicine
P60709	ACTB	Actin, beta	Cytoplasm	Other	
P14136	GFAP	Glial fibrillary acidic protein	Cytoplasm	Other	
Q562R1	ACTBL2	Actin, beta-like 2	Nucleus	Other	
P07195	LDHB	Lactate dehydrogenase B	Cytoplasm	Enzyme	
P62258	YWHAE	Tyrosine 3-monooxygenase/tryptophan 5-monooxygenase activation protein, epsilon	Cytoplasm	Other	
P08758	ANXA5	Annexin A5	Plasma Membrane	Other	
P02647	APOA1	Apolipoprotein A-I	Extracellular Space	Transporter	
P06733	ENO1	Enolase 1, (alpha)	Cytoplasm	Enzyme	
Q13332	PTPRS*	Protein tyrosine phosphatase, receptor type, S	Plasma Membrane	Phosphatase	
Q13332	PTPRS*	Protein tyrosine phosphatase, receptor type, S	Plasma Membrane	Phosphatase	
Q96P48	ARAP1	ArfGAP with RhoGAP domain, ankyrin repeat and PH domain 1	Cytoplasm	Other	
Q8TEP8	CEP192	Centrosomal protein 192kDa	Cytoplasm	Other	
Q9Y4D7	PLXND1	Plexin D1	Plasma Membrane	Transmembrane receptor	
Q9P2D3	HEATR5B	HEAT repeat containing 5B	Other	Other	
Q5MCW4	ZNF569	Zinc finger protein 569	Nucleus	Other	

P06865	HEXA	Hexosaminidase A (alpha polypeptide)	Cytoplasm	Enzyme
P49639	HOXA1	Homeobox A1	Nucleus	Transcription regulator
Q6P2Q9	PRPF8	Pre-mRNA processing factor 8	Nucleus	Other

Supplemental Table 3. Signaling pathway networks that involve phosphotyrosine-containing proteins identified from human glioblastoma.

Networks	Nodes (genes; proteins) in Network	Score	Nodes	Identified nodes (pY-proteins)	Top Diseases and Functions
1	14-3-3, ACTB, Akt, Alpha tubulin, ANXA5, APOA1, Ck2, DNAJC24, DNAJC25, ENO1, ERK, ERK1/2, GFAP, HOXA1, HSP, Hsp70, Hsp90, HSP90AA1, HSP90AB1, HSPA1A/HSPA1B, Insulin, Jnk, NFkB (complex), P38 MAPK, PRPF8, PTPRS, RNA polymerase II, Rock, TRAP1, TUBA8, TUBA1A, TUBA1B, tubulin (complex), tubulin (family), YWHAE	43	36	16	Cancer, Organismal Injury and Abnormalities, Reproductive System Disease
2	ACTBL2, ARAP1, CD1E, CEP85, CEP192, CHI3L2, DCHS1, EFR3A, GPR126, GPR176, HEATR5B, HEXA, HPS1, LDHAL6A, LDHAL6B, LDHB, MRPS33, NLRC3, PLEK2, PLXND1, PSD3, PXMP2, RAB37, RABL2B, RBM12B, RNASE7, SLC16A5, SLC43A2, SPATA2L, SYNGR3, TMEM115, TNF, UBC, UTP11L, ZNF330	15	35	7	Cell Morphology, Cellular Assembly and Organization, Cellular Function and Maintenance
3	CBX5, CDC37, ZNF569	3	3	1	Cancer, Organismal Injury and Abnormalities, Developmental Disorder

Supplemental Figure 1

Significant canonical pathways involved phosphotyrosine-containing proteins in a glioblastoma tissue

1. 14-3-3-mediated signaling
2. Aldosterone signaling in epithelial cells
3. Aryl hydrocarbon receptor signaling
4. Axonal guidance signaling
5. Breast cancer regulation by stathmin1
6. Cell cycle G2/M DNA damage checkpoint regulation
7. Chondroitin sulfate degradation (Metazoa)
8. Clathrin-mediated endocytosis signaling
9. Dematan sulfate degradation (Metazoa)
10. eNOS signaling
11. Epithelial adherens junction signaling
12. Gap junction signaling
13. Germ cell-sertoli cell junction signaling
14. Glucocorticoid receptor signaling
15. Gluconeogenesis I
16. Glycolysis I
17. HIF1a signaling
18. Hypoxia signaling in the cardiovascular system
19. Mechanisms of viral exit from host cells
20. Mitotic roles of Polo-like kinase
21. MSP-RON signaling pathway
22. Neuregulin signaling
23. Neuroprotective role of THOP1 in Alzheimer's Disease
24. Nitric oxide signaling in the cardiovascular system
25. PI3K-AKT signaling
26. PPAR signaling
27. PPARa-RXRa activation
28. Prostate cancer signaling
29. Protein ubiquitination pathway
30. Pyruvate fermentation to lactate
31. Remodeling of epithelial adherens junctions
32. Sertoli cell-sertoli cell junction signaling
33. Signaling by Rho family GTPases
34. Telomerase signaling
35. VEGF signaling
36. Xenobiotic metabolism signaling

1. 14-3-3-mediated signaling

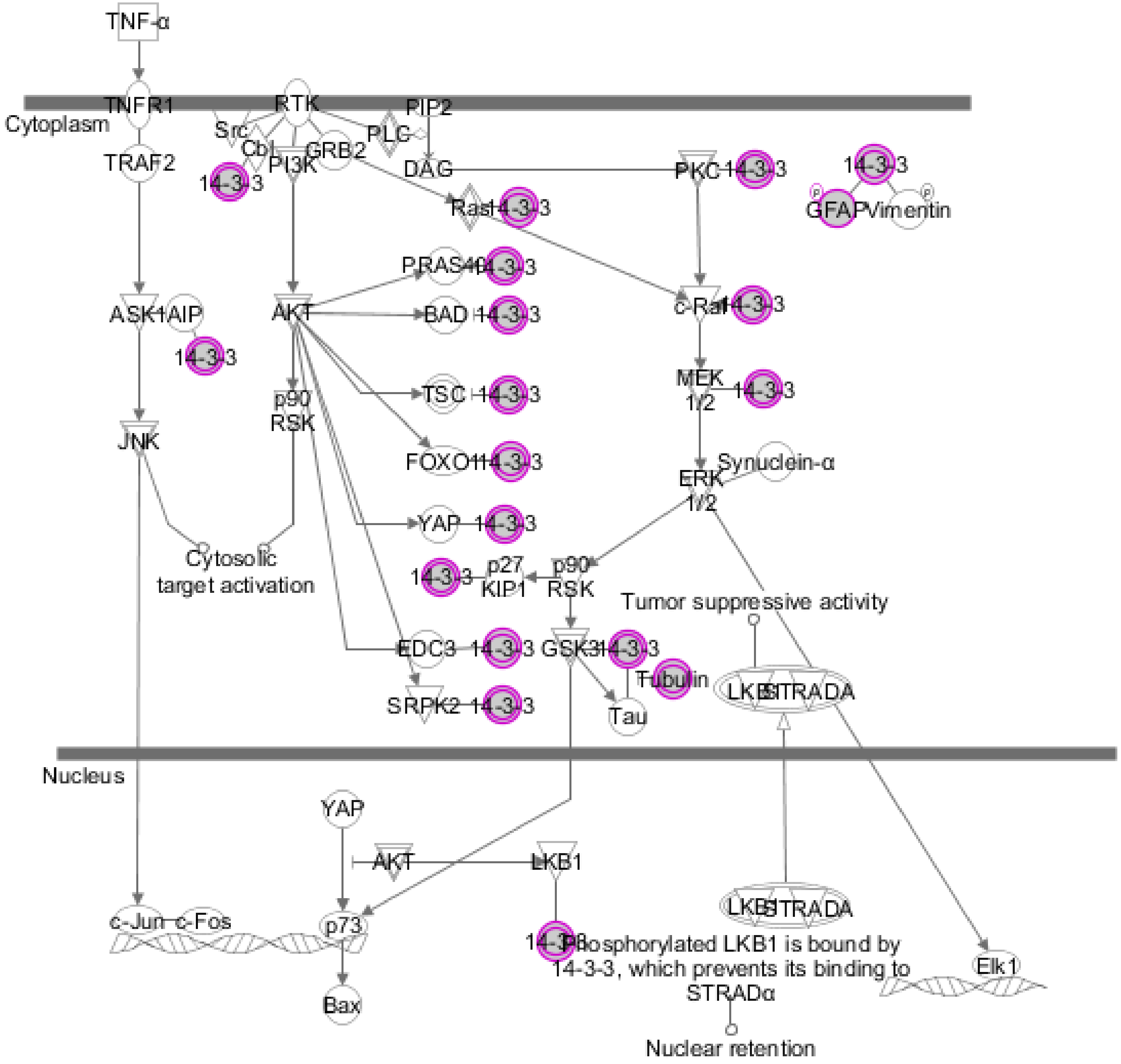

2. Aldosterone signaling in epithelial cells

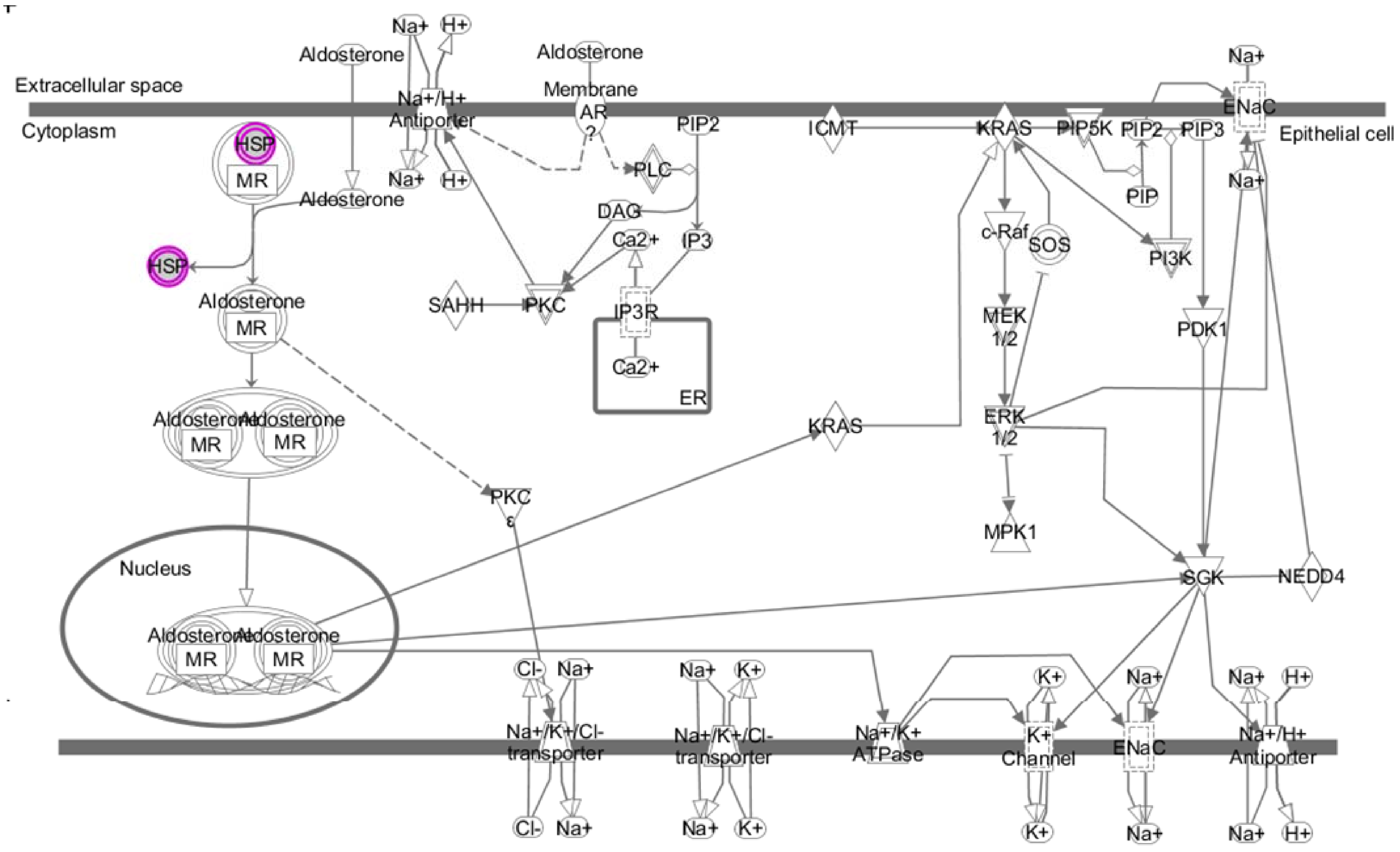

3. Aryl hydrocarbon receptor signaling

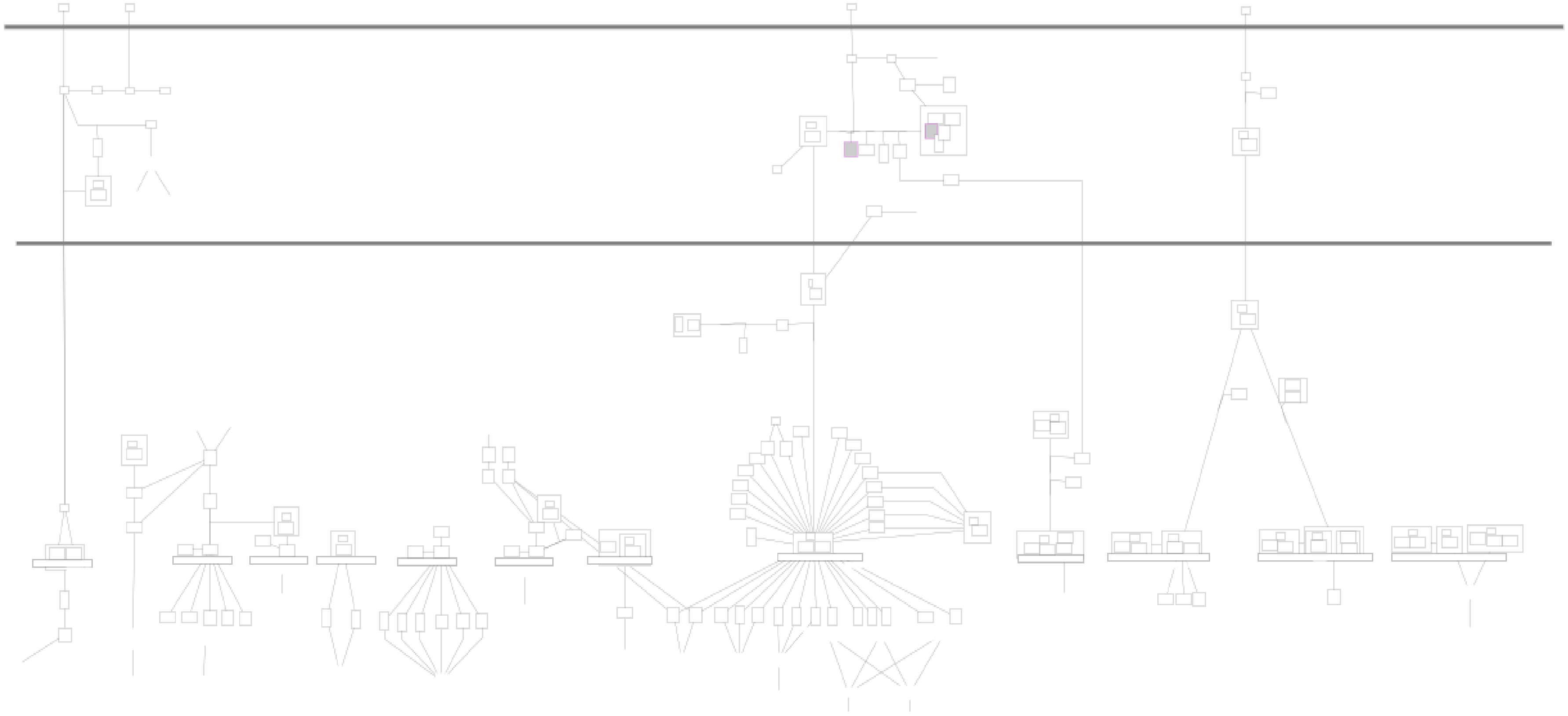

4. Axonal guidance signaling

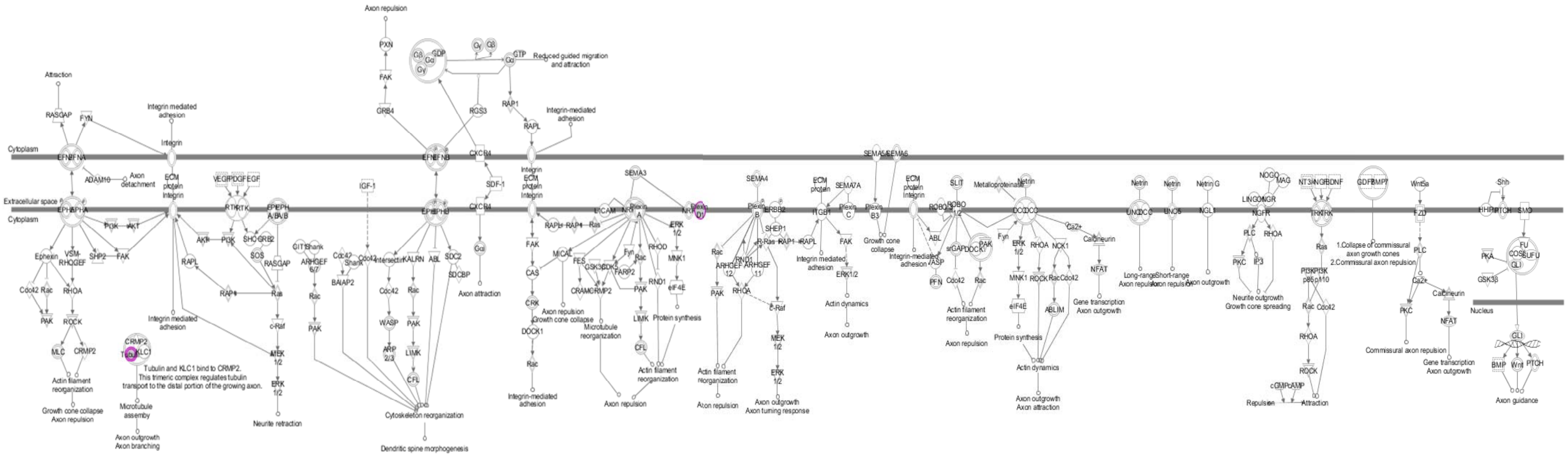

5. Breast cancer regulation by stathmin1

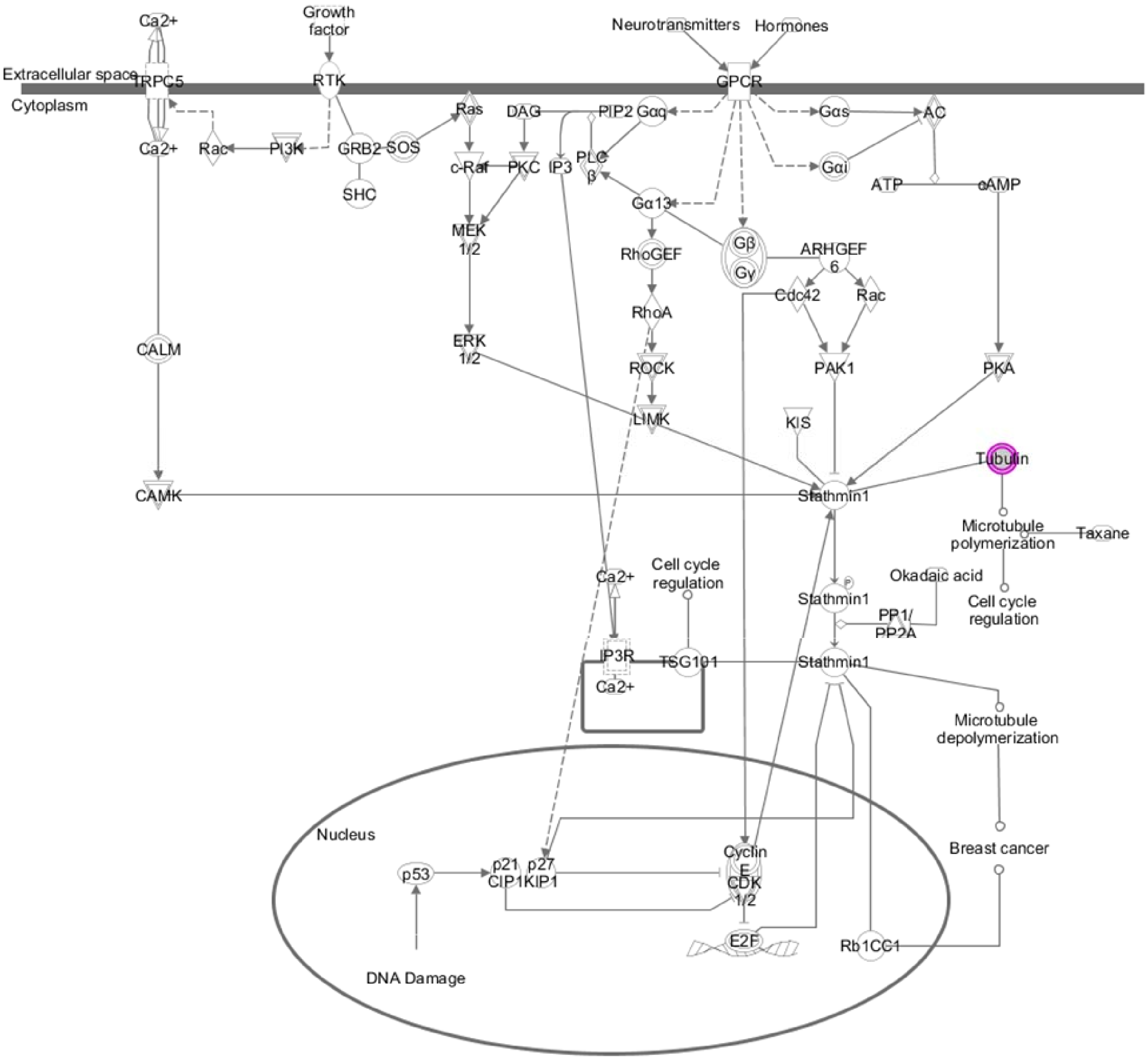

6. Cell cycle G2/M DNA damage checkpoint regulation

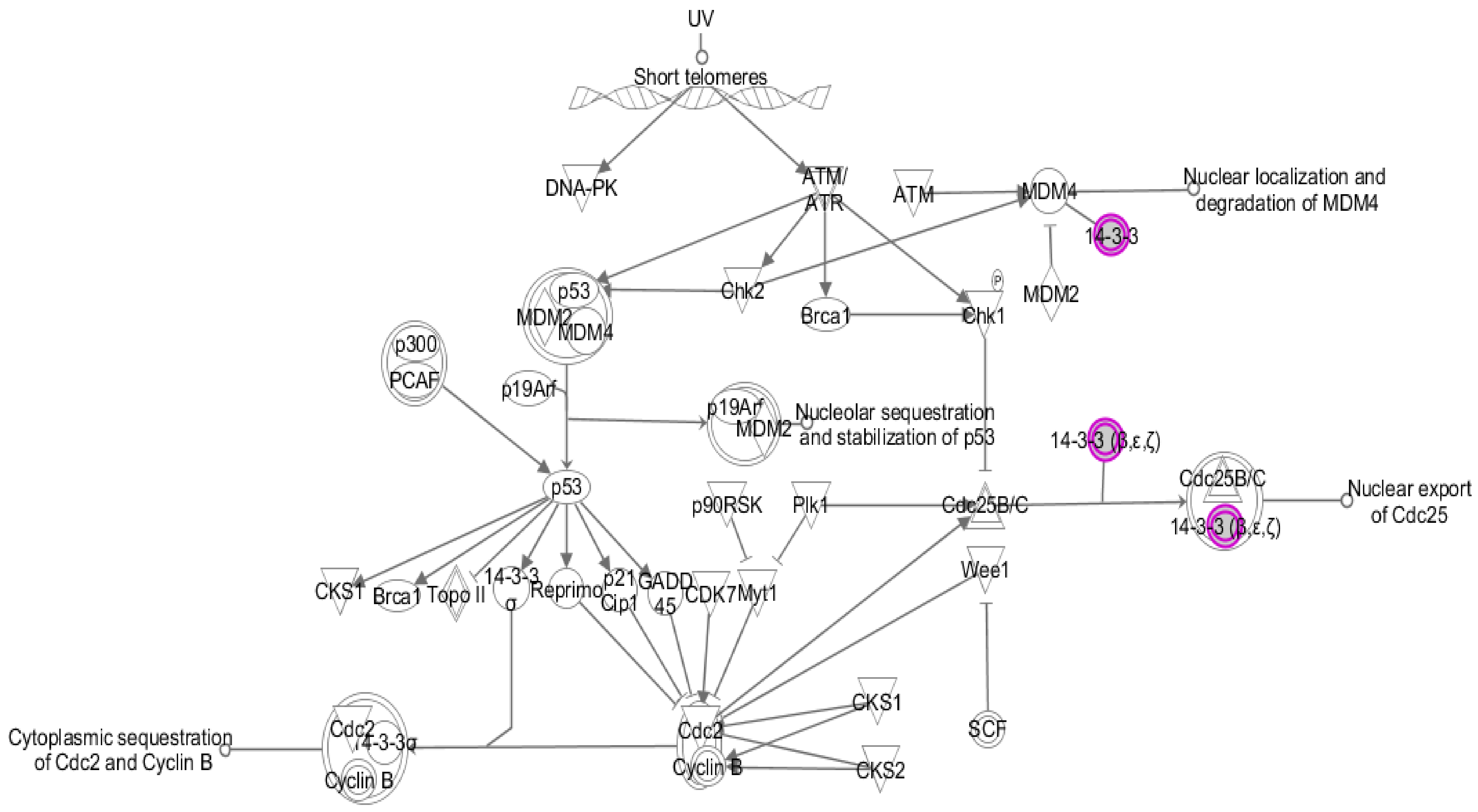

7. Chondroitin sulfate degradation (Metazoa)

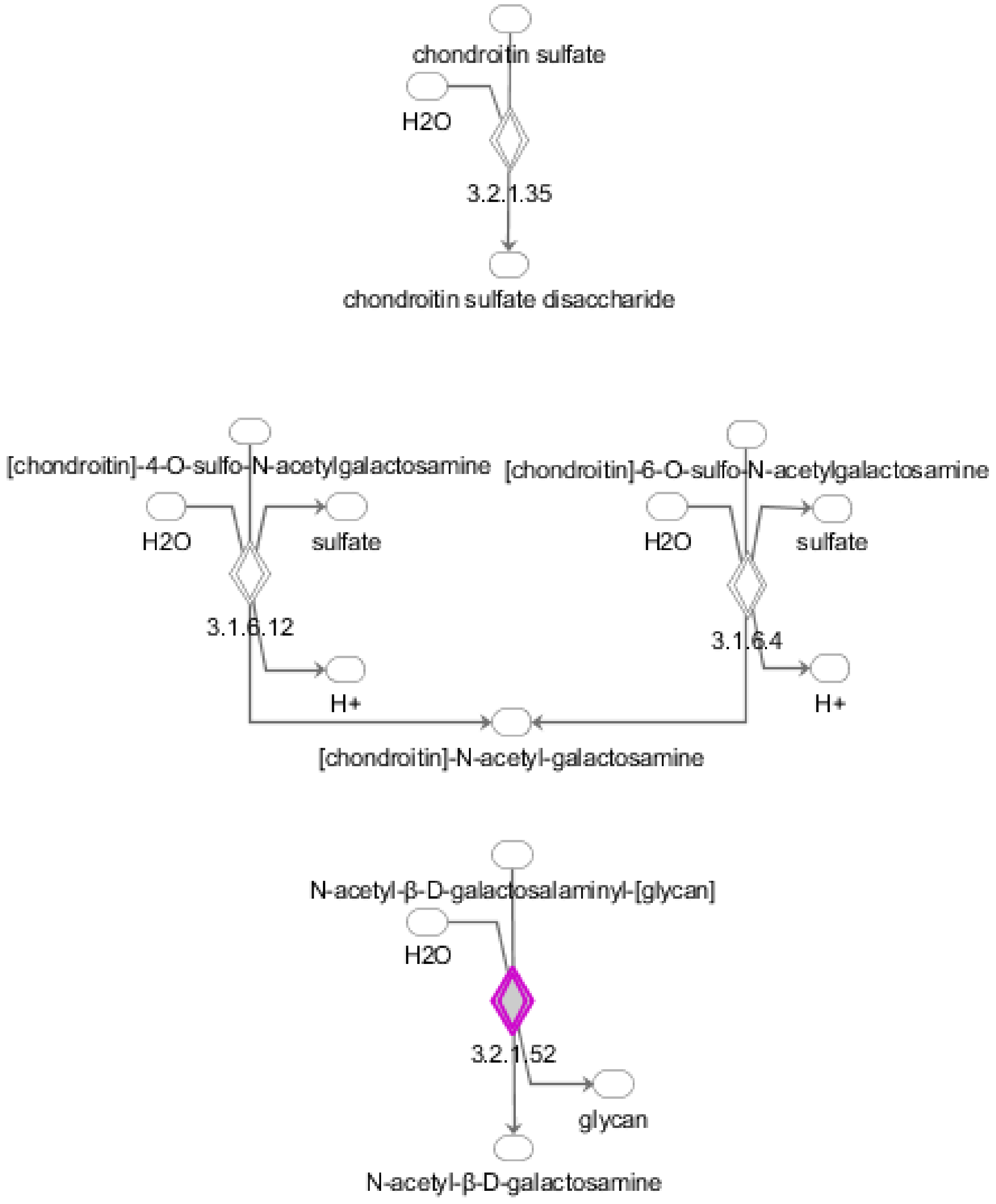

8. Clathrin-mediated endocytosis signaling

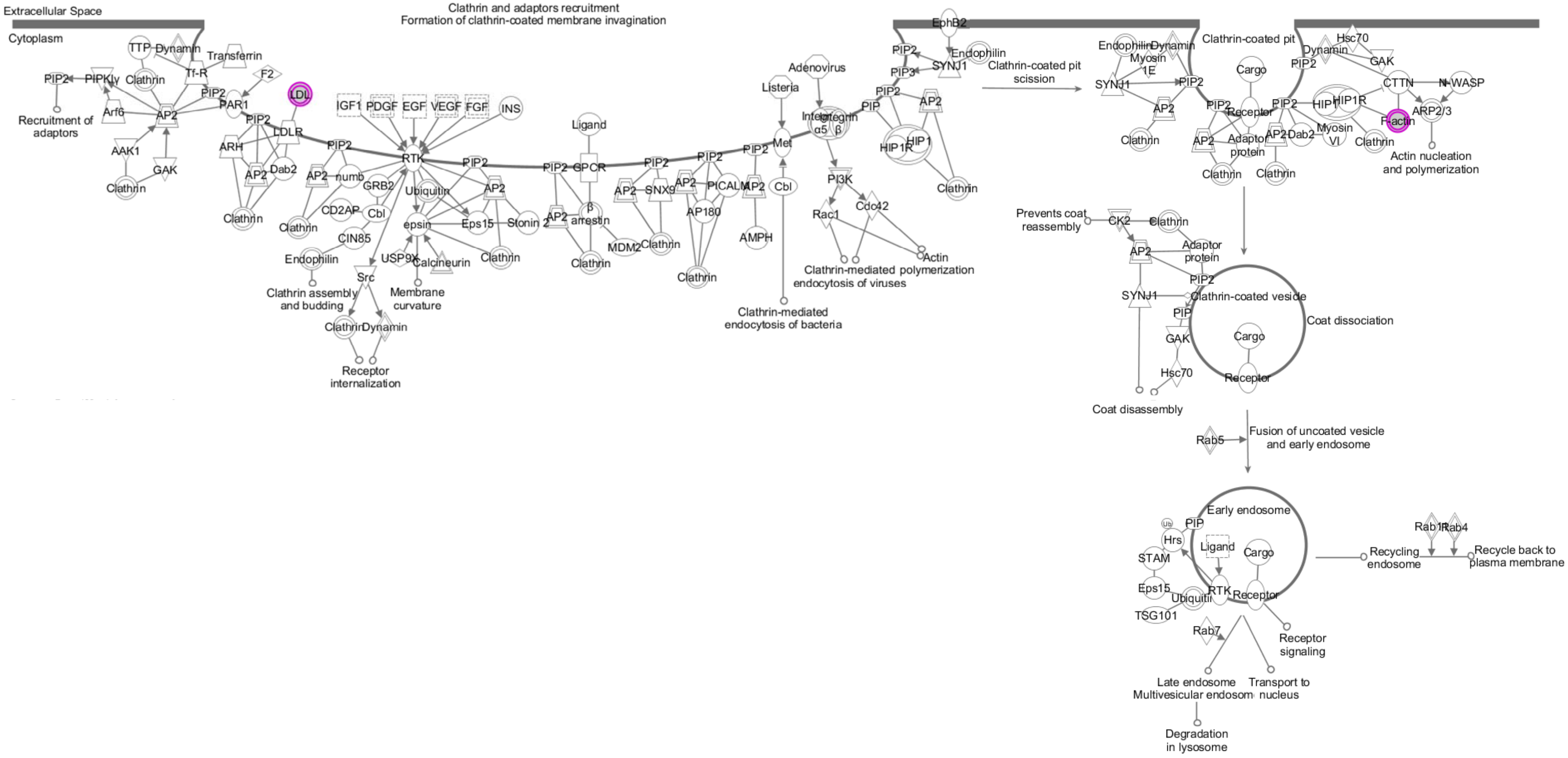

9. Dematan sulfate degradation (Metazoa)

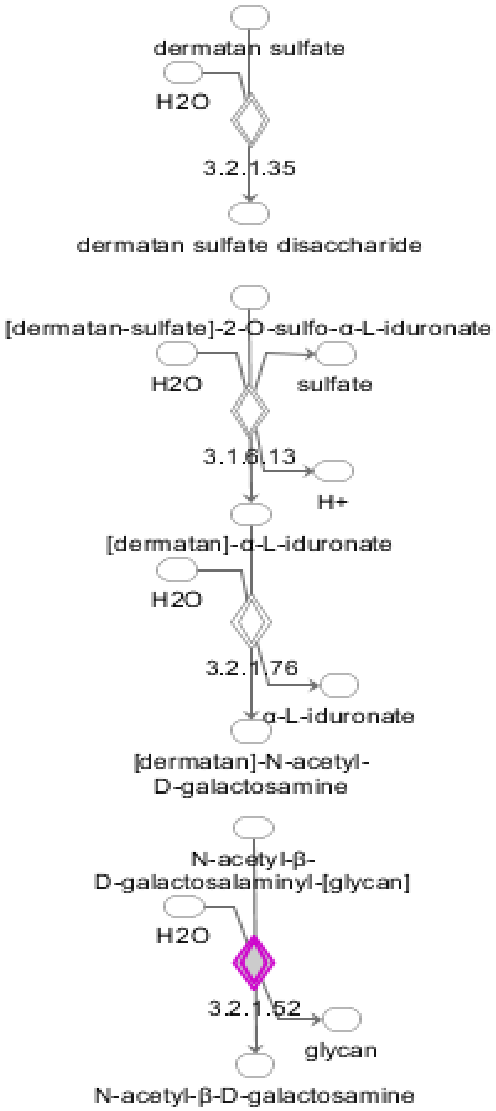

10. eNOS signaling

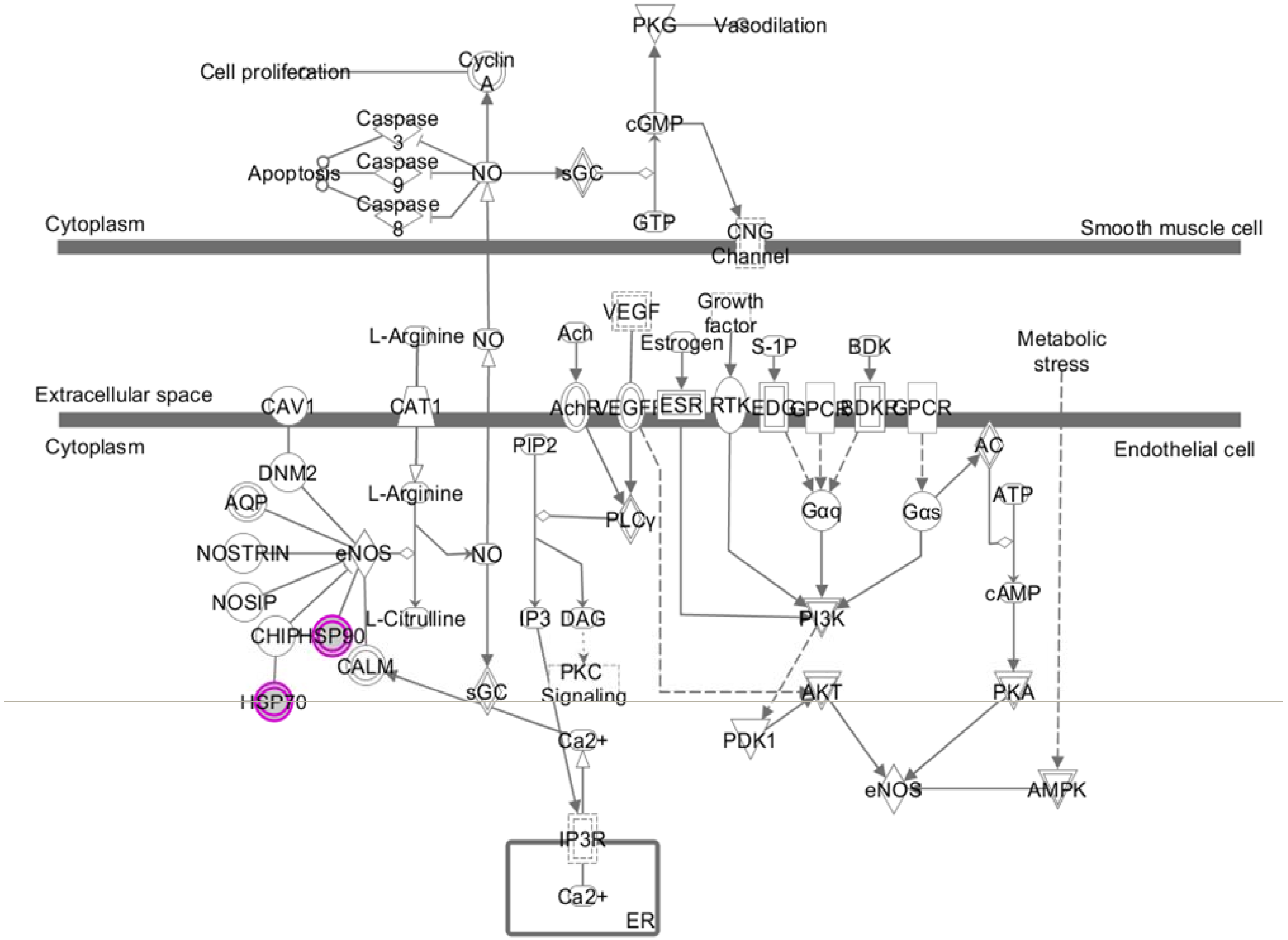

11. Epithelial adherens junction signaling

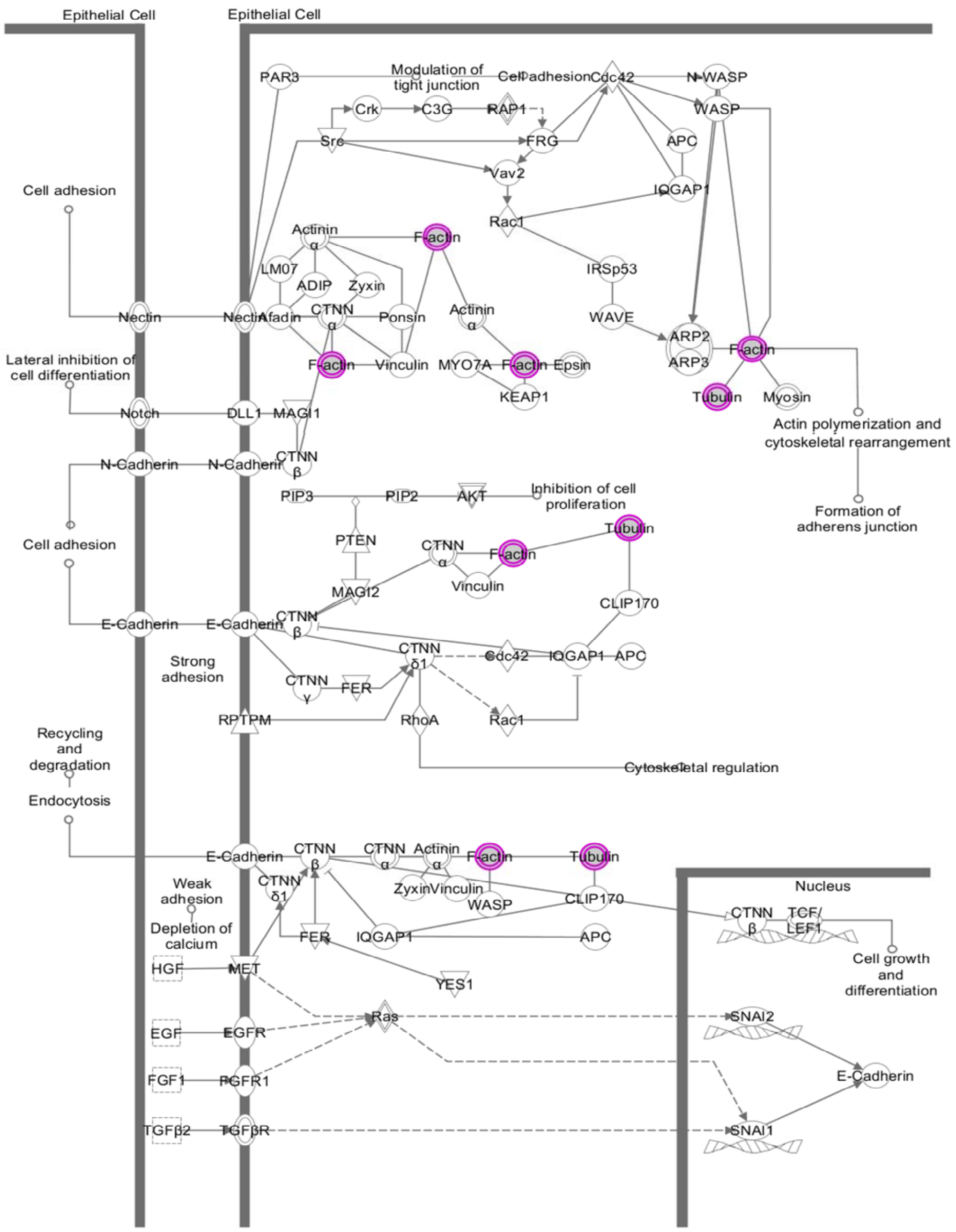

12. Gap junction signaling

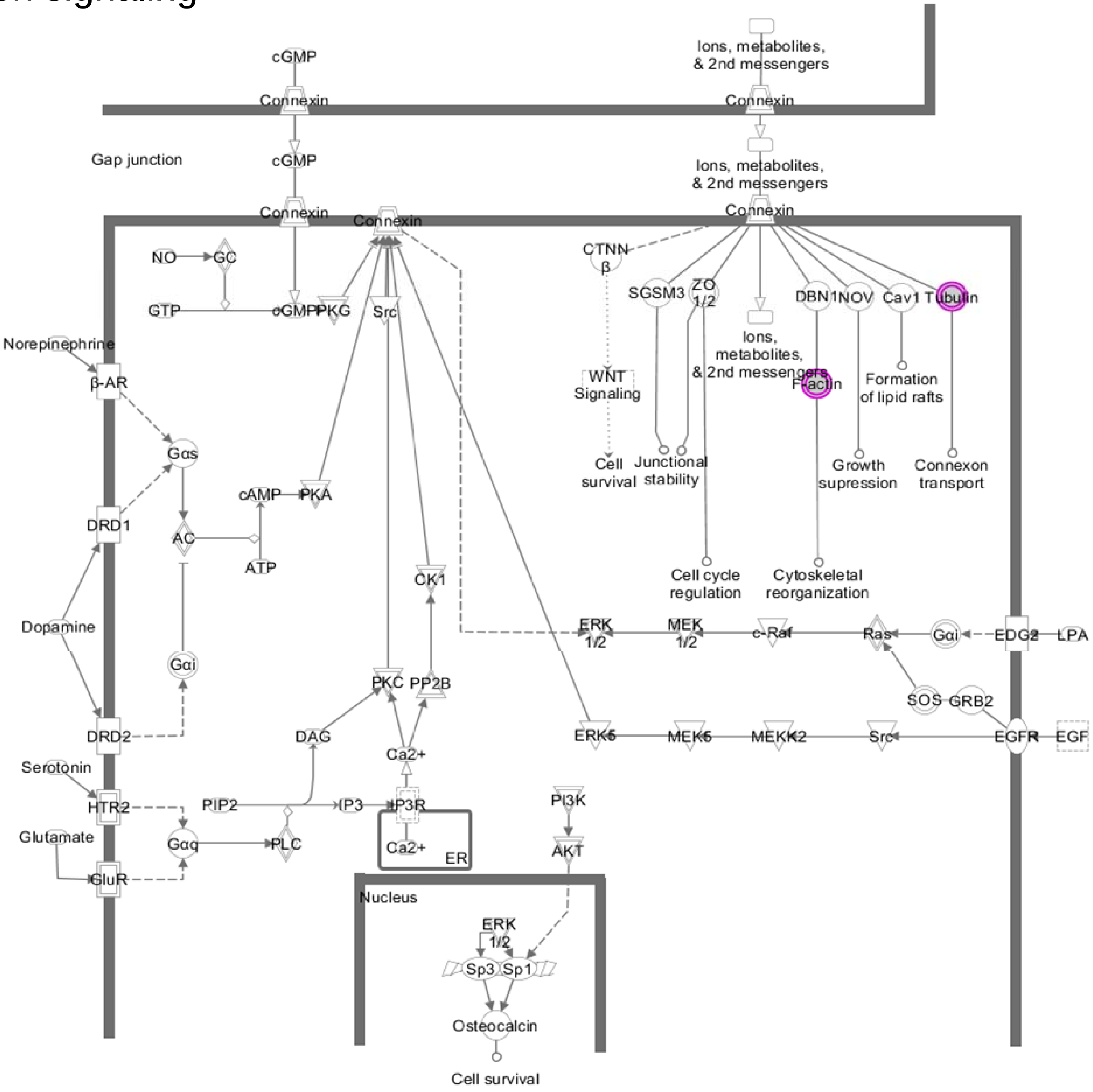

13. Germ cell-sertoli cell junction signaling

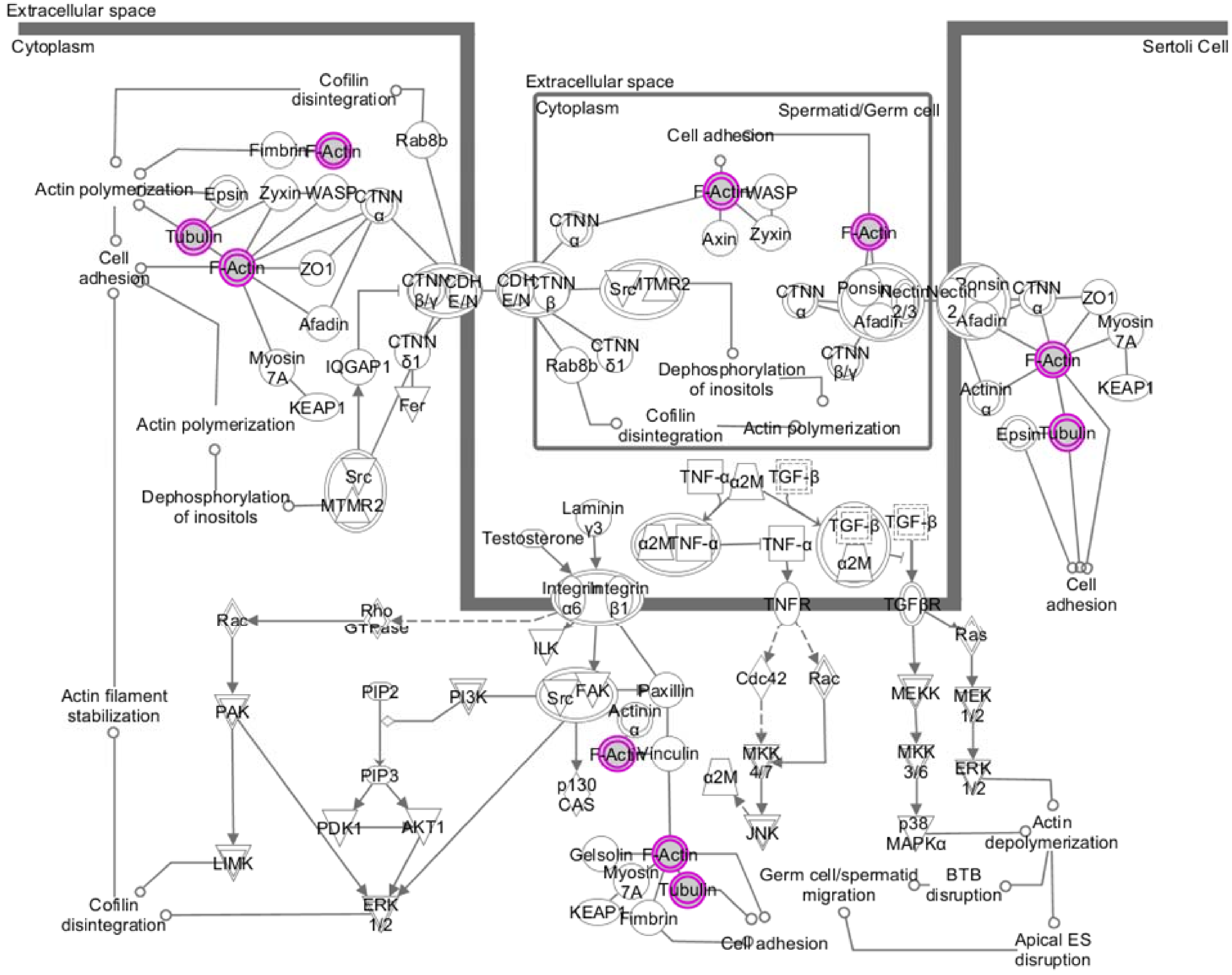

14. Glucocorticoid receptor signaling

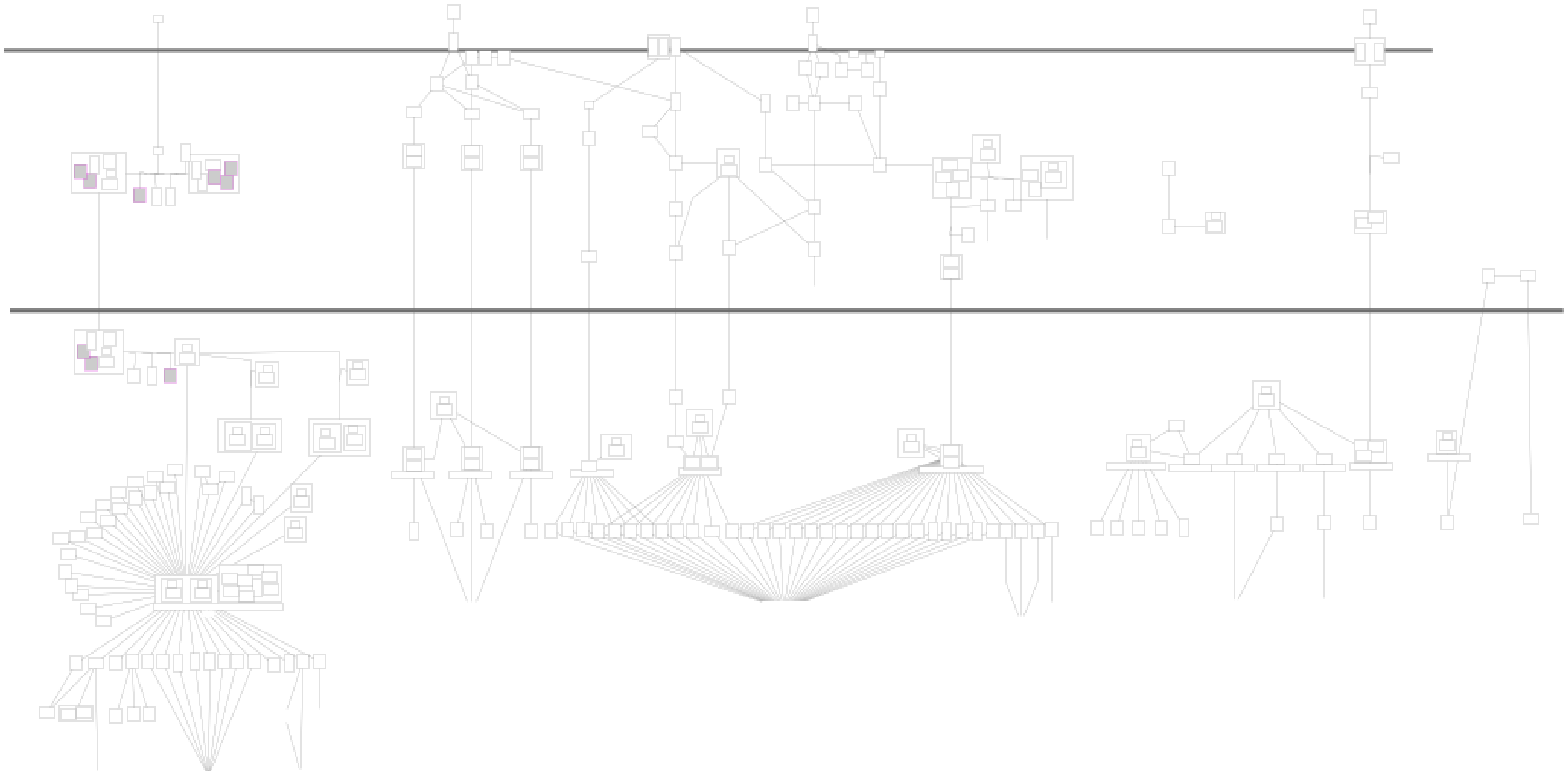

15. Gluconeogenesis I

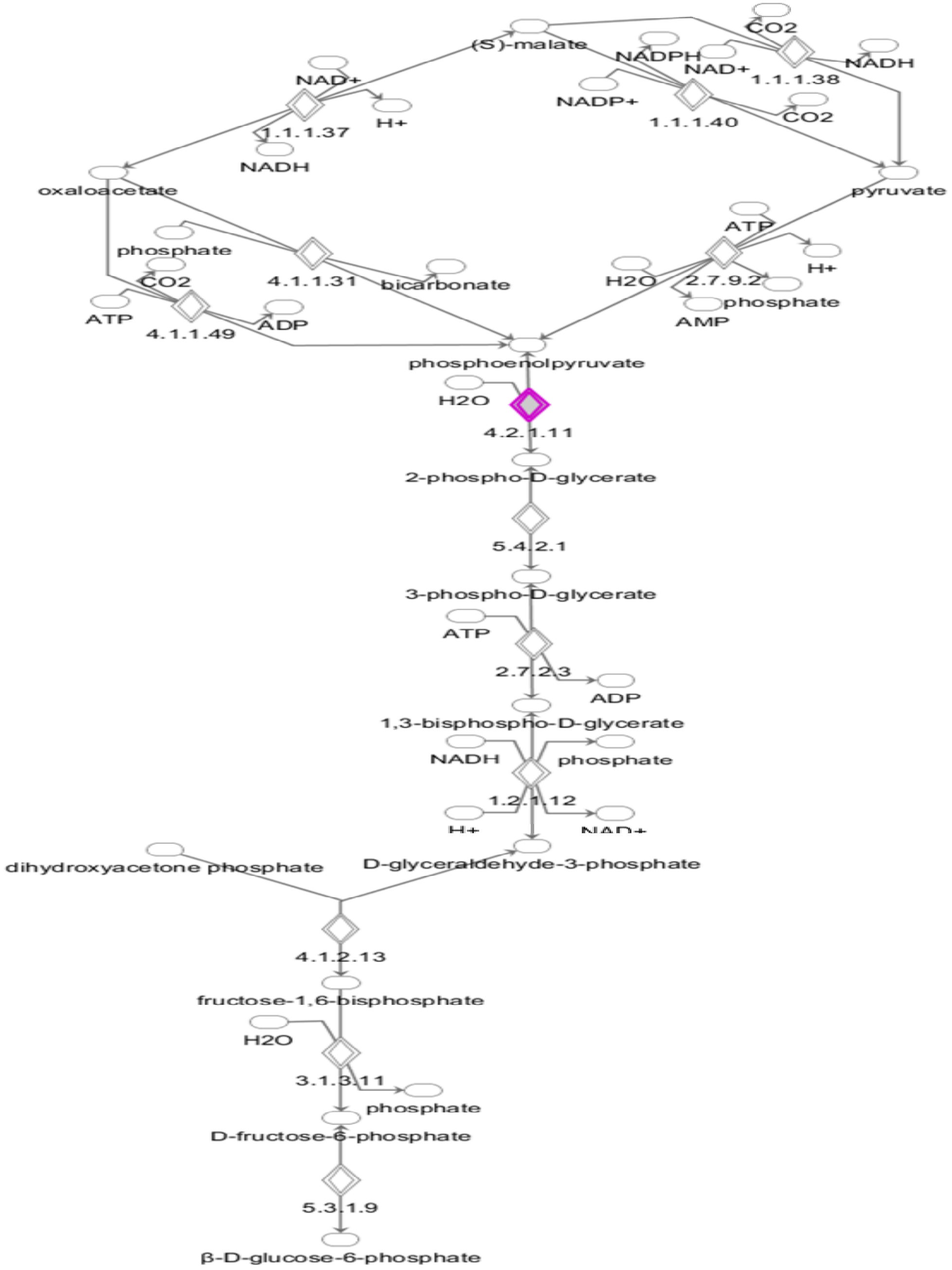

16. Glycolysis I

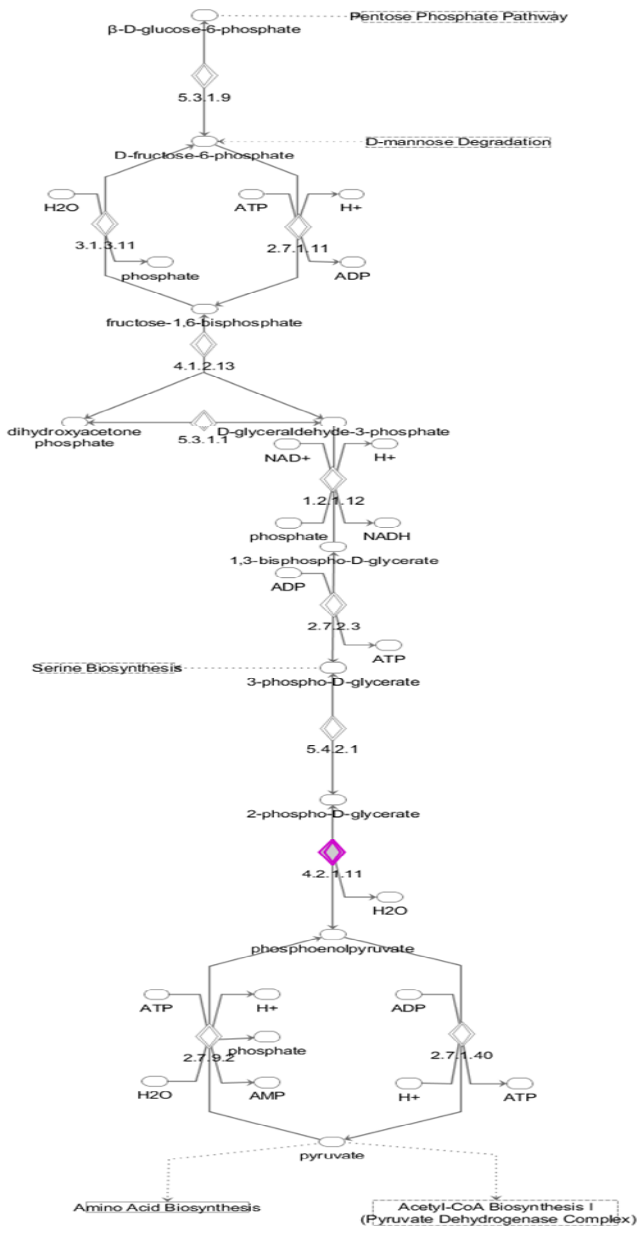

17. HIF1a signaling

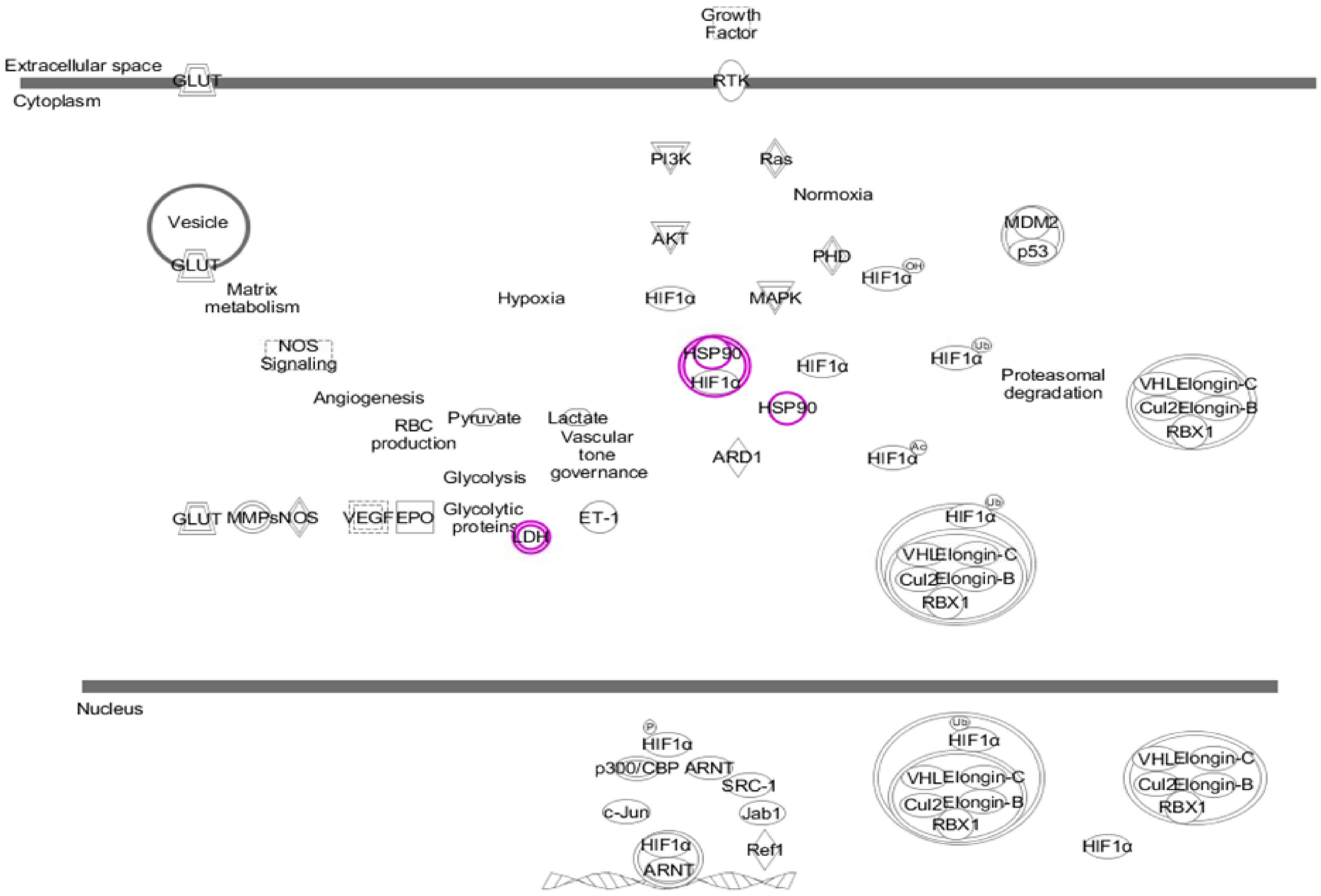

18. Hypoxia signaling in the cardiovascular system

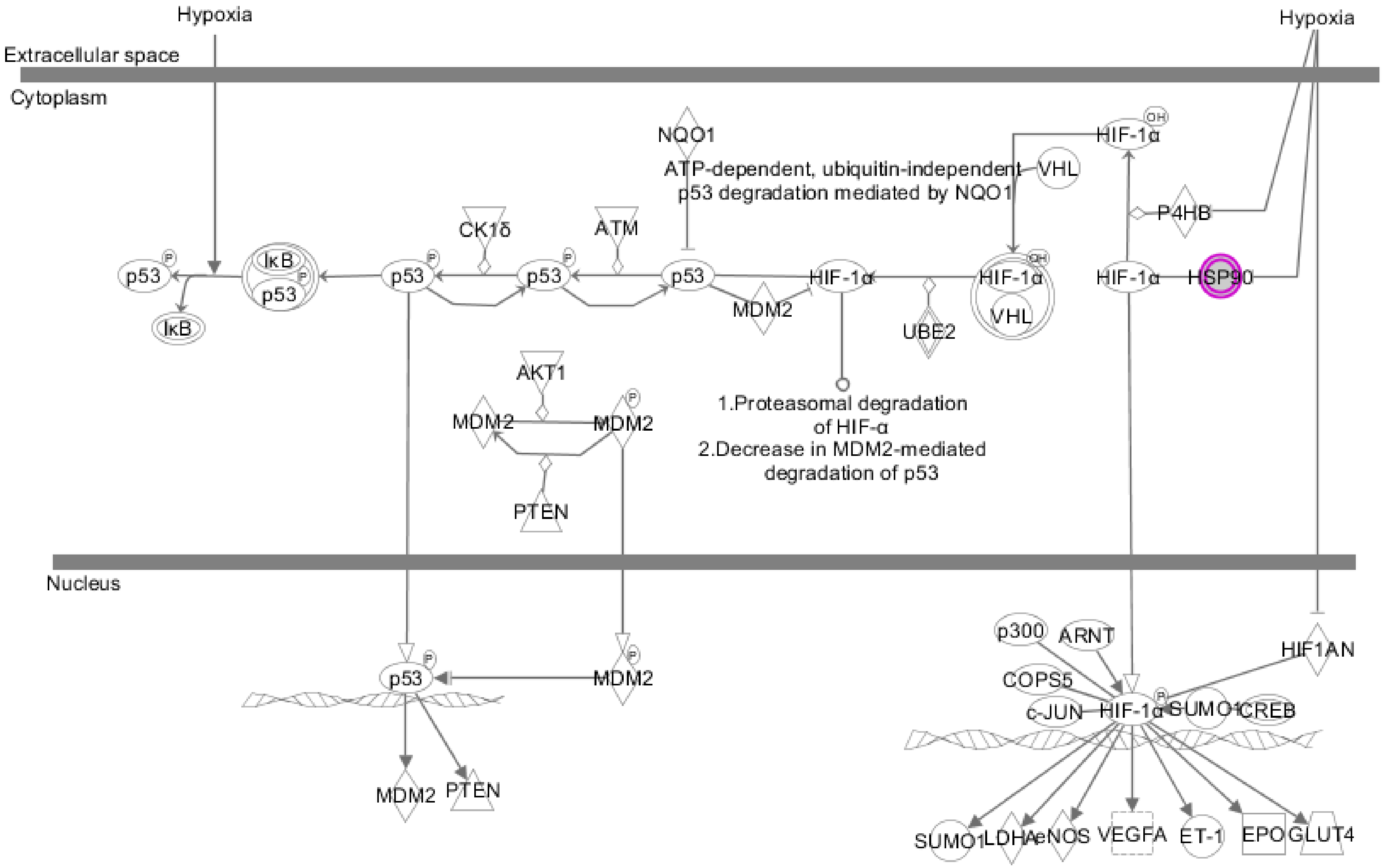

19. Mechanisms of viral exit from host cells

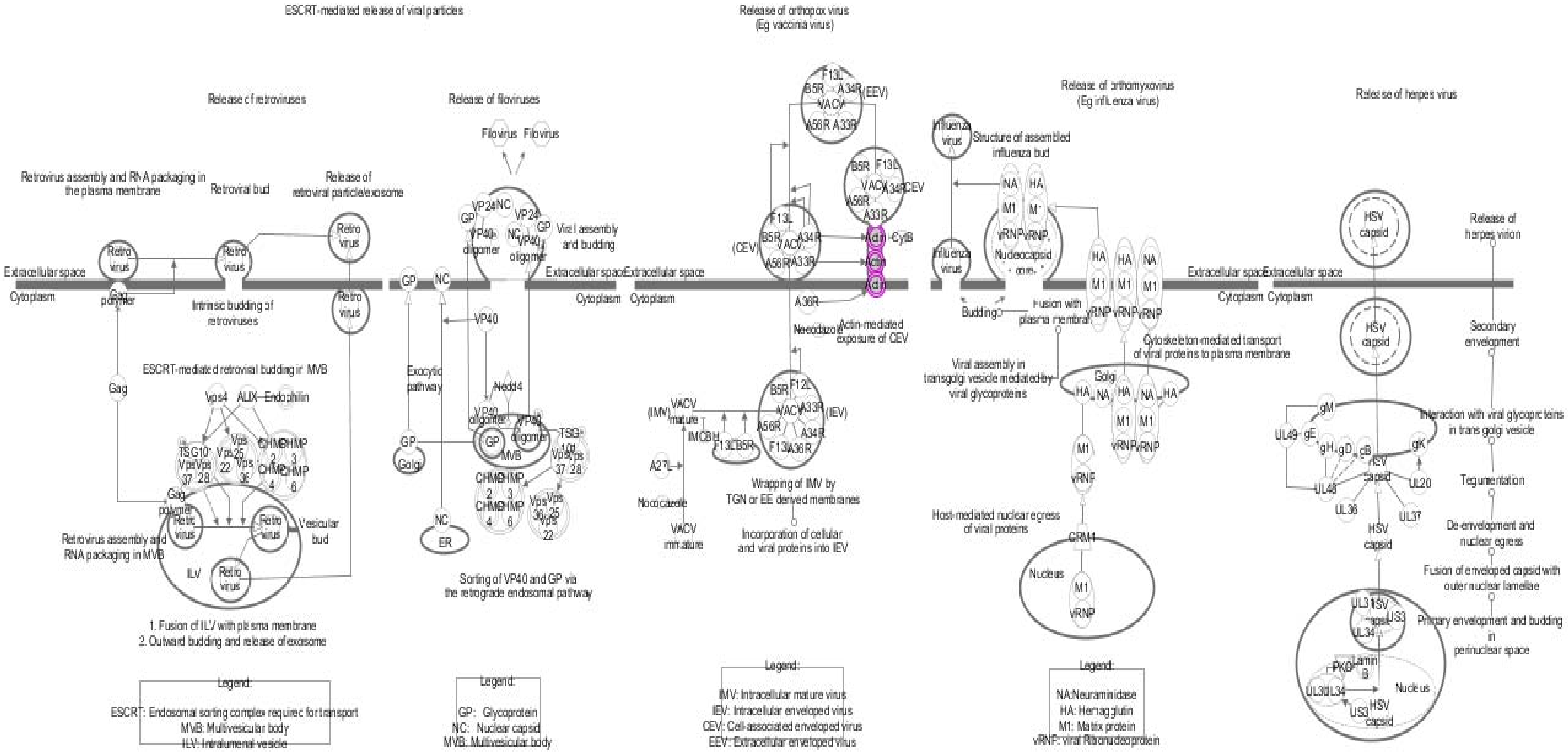

20. Mitotic roles of Polo-like kinase

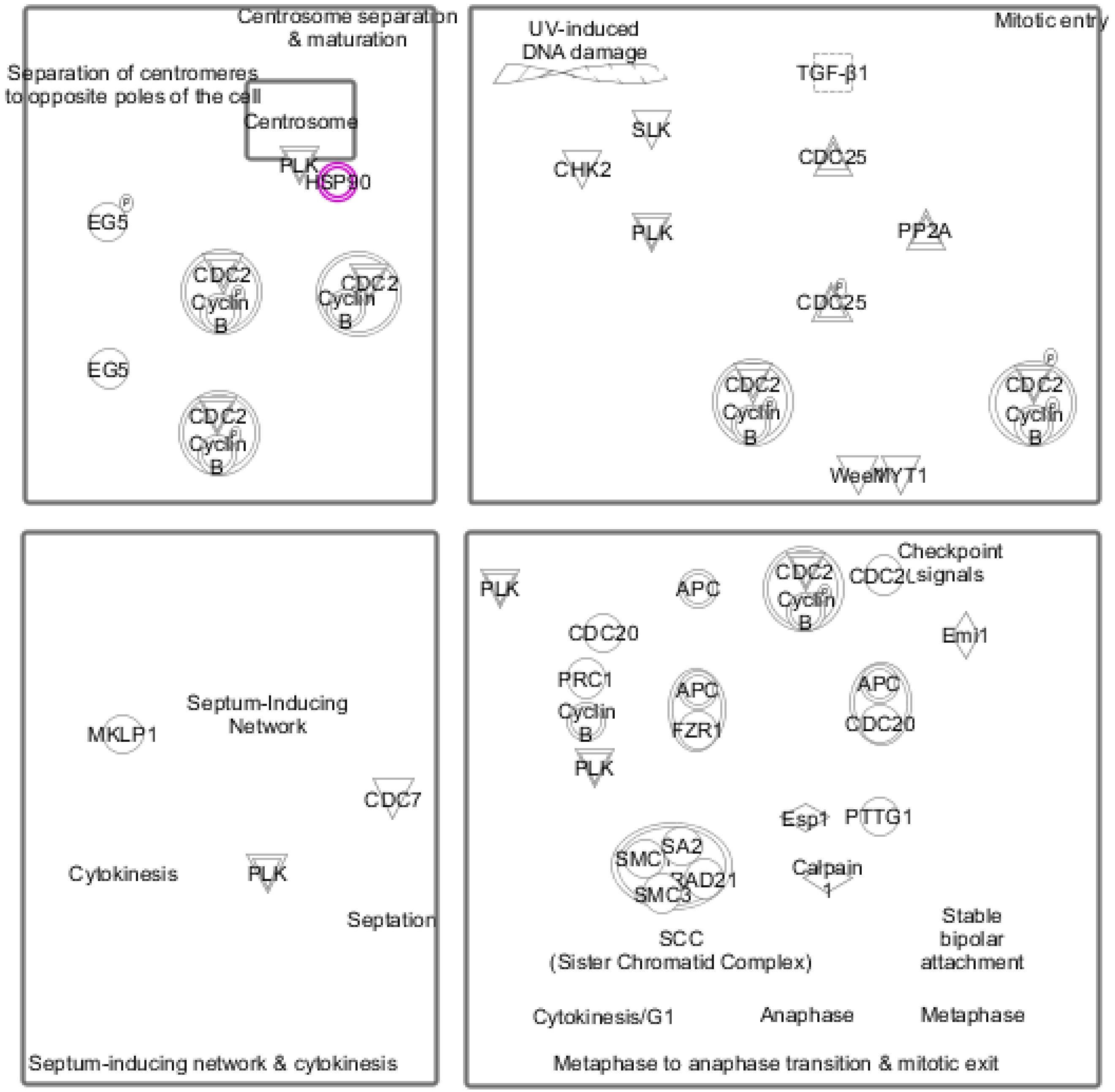

21. MSP-RON signaling pathway

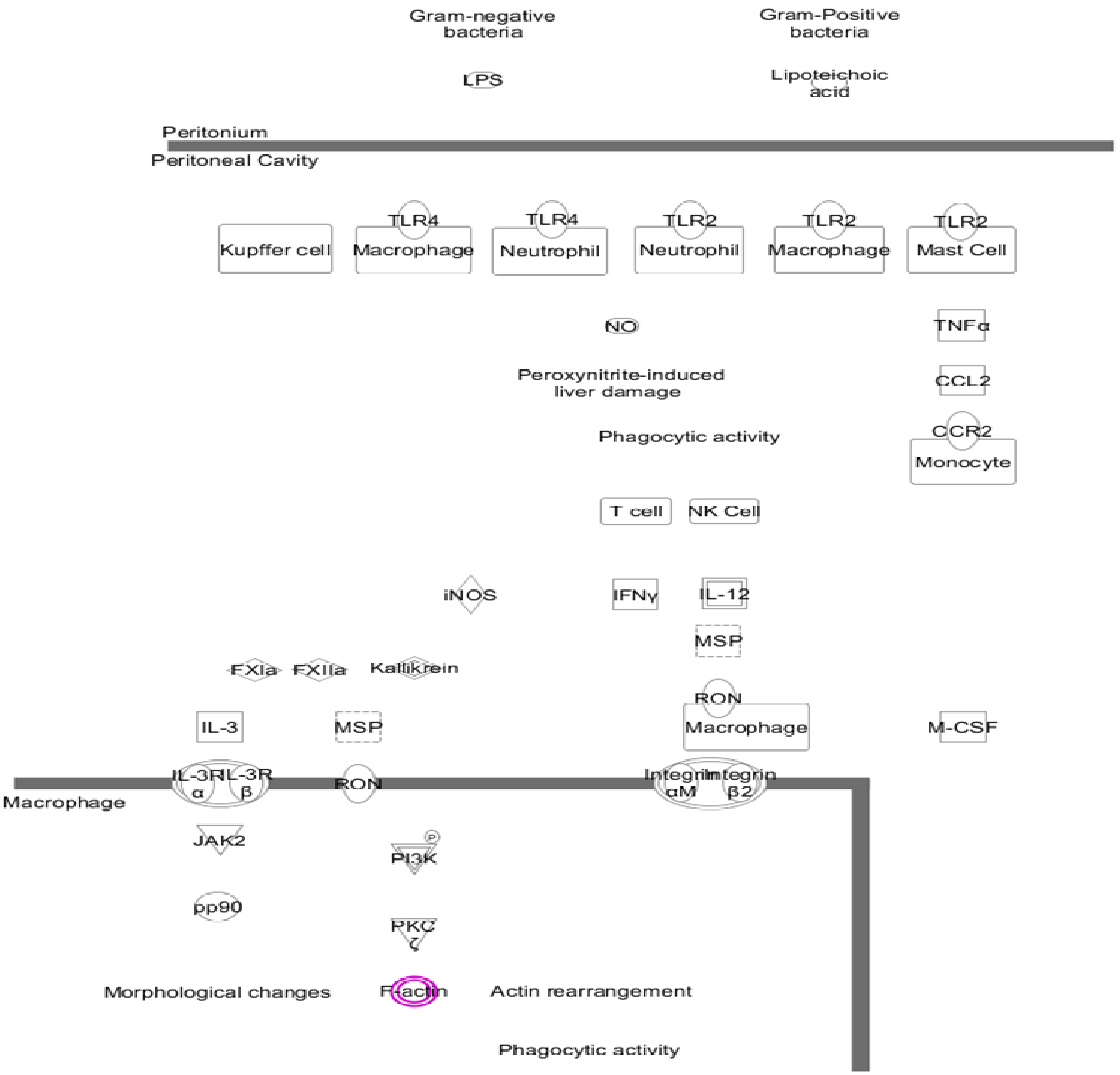

22. Neuregulin signaling

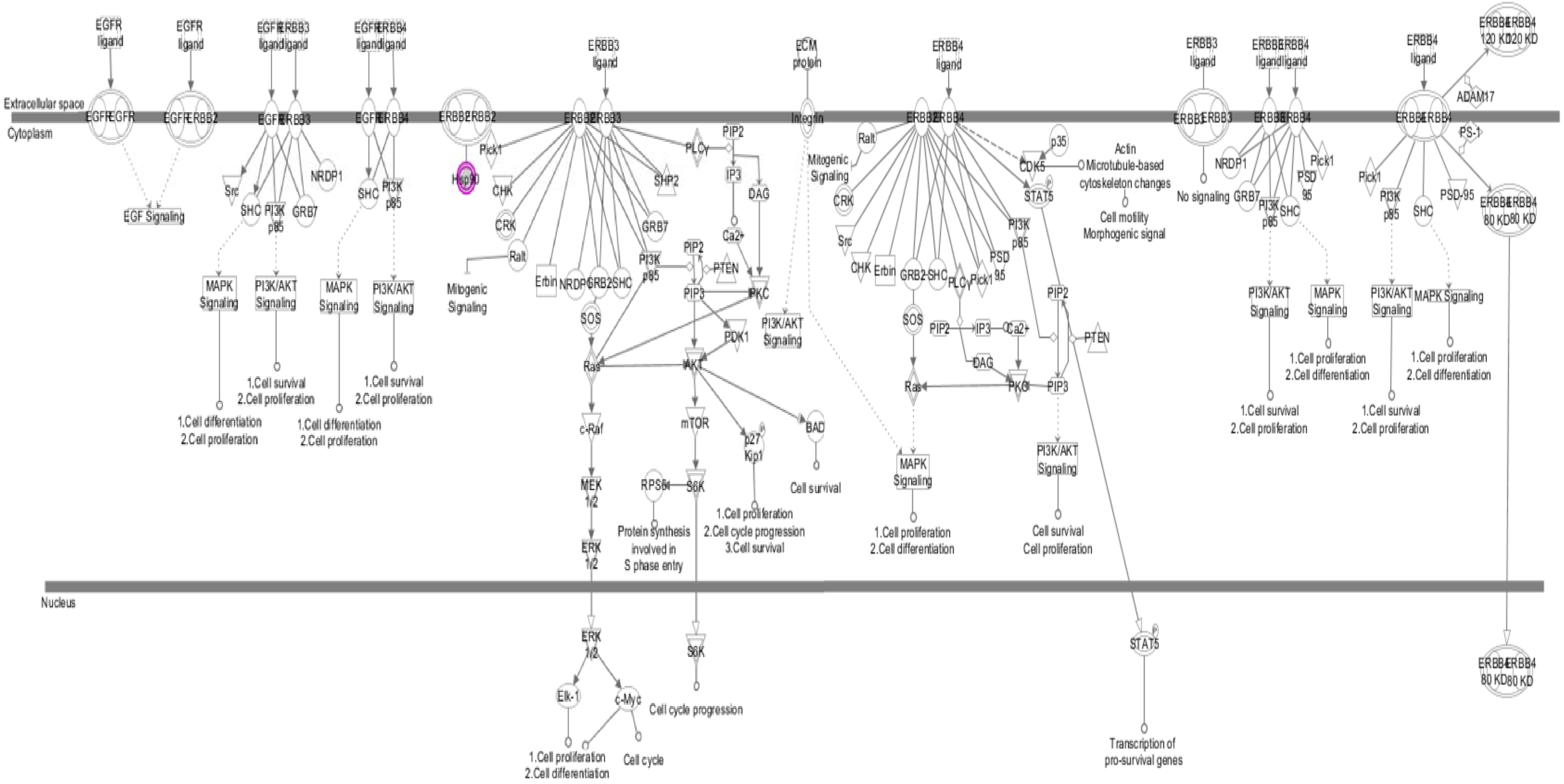

23. Neuroprotective role of THOP1 in Alzheimer's Disease

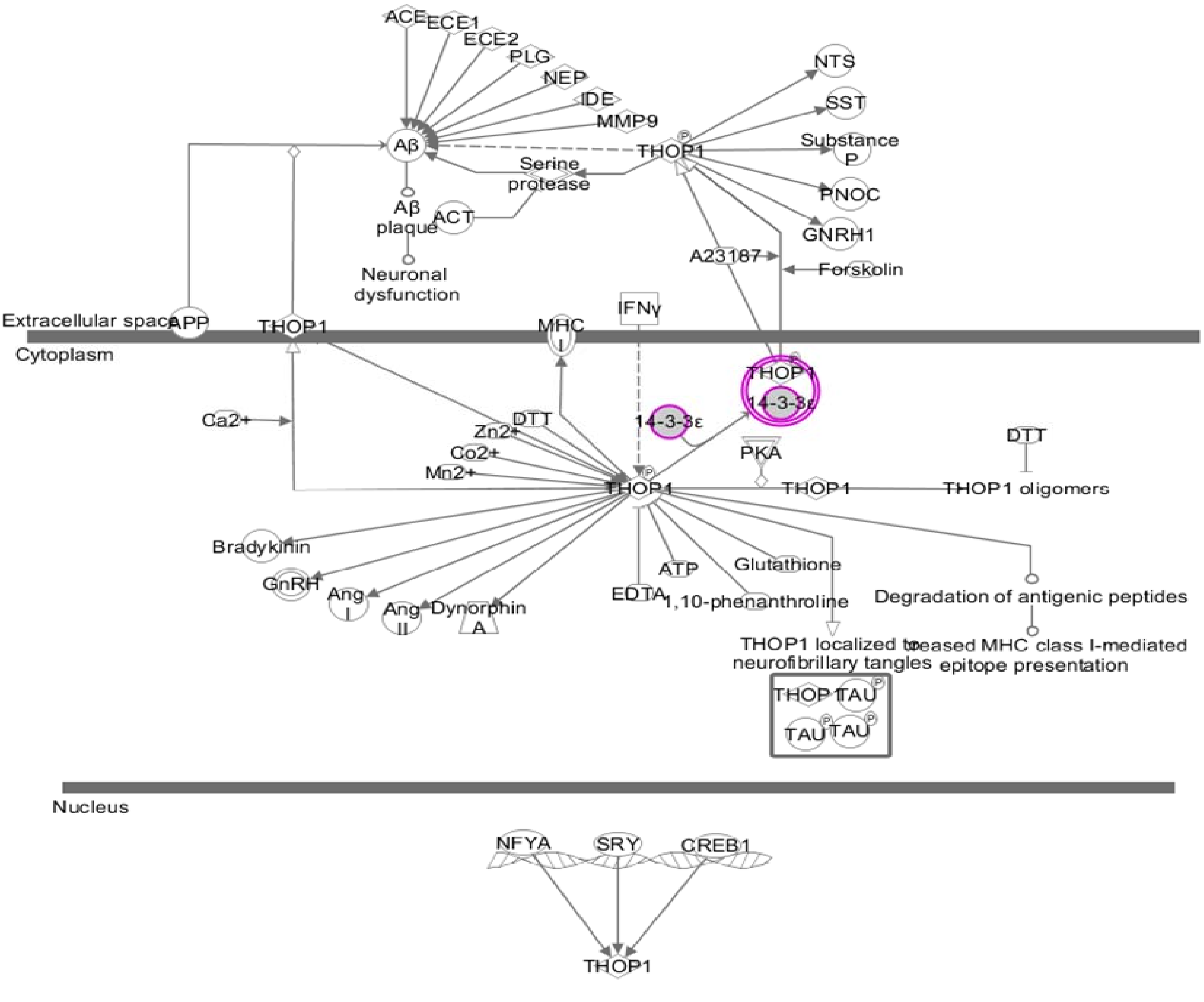

24. Nitric oxide signaling in the cardiovascular system

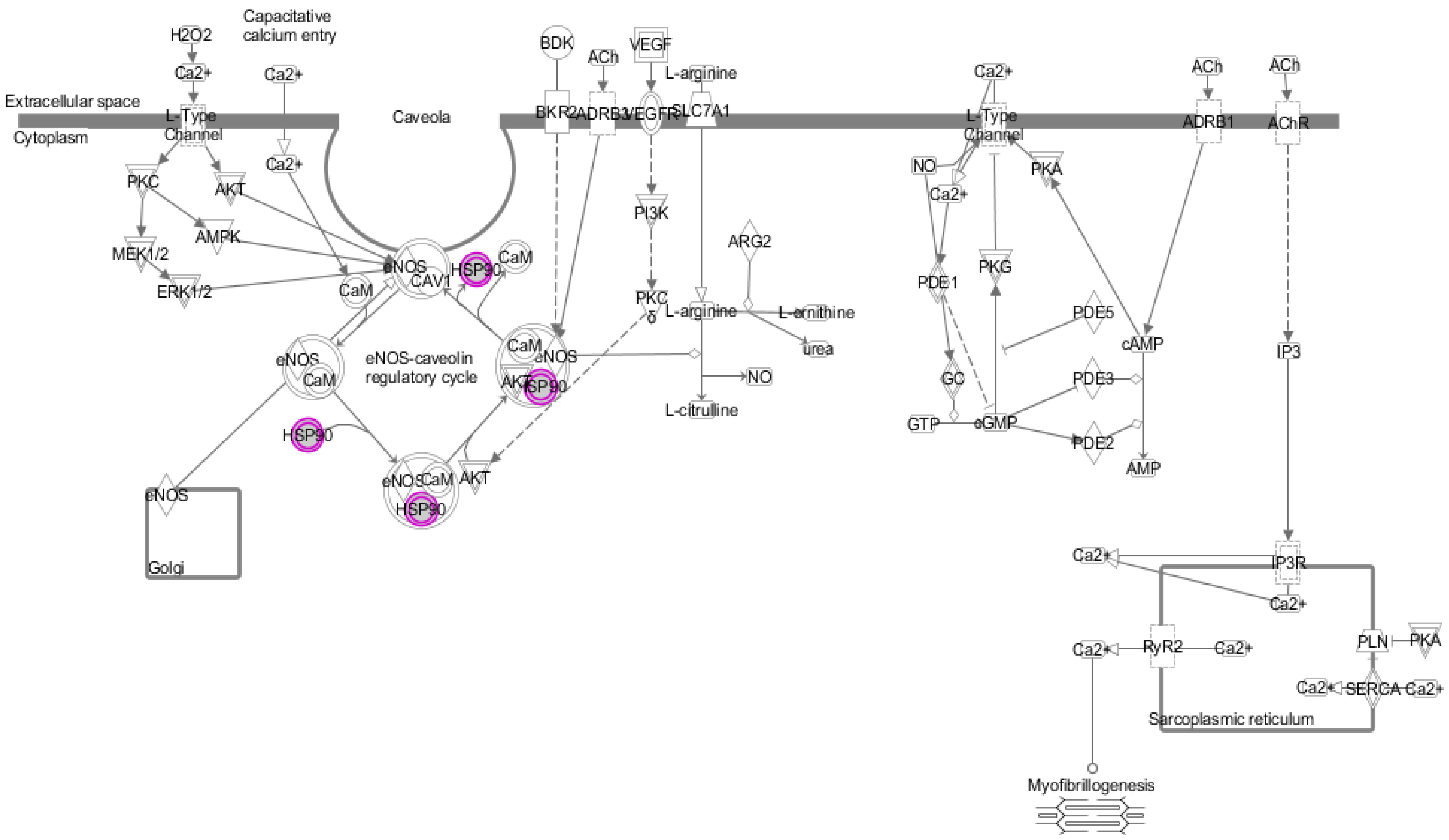

25. PI3K-AKT signaling

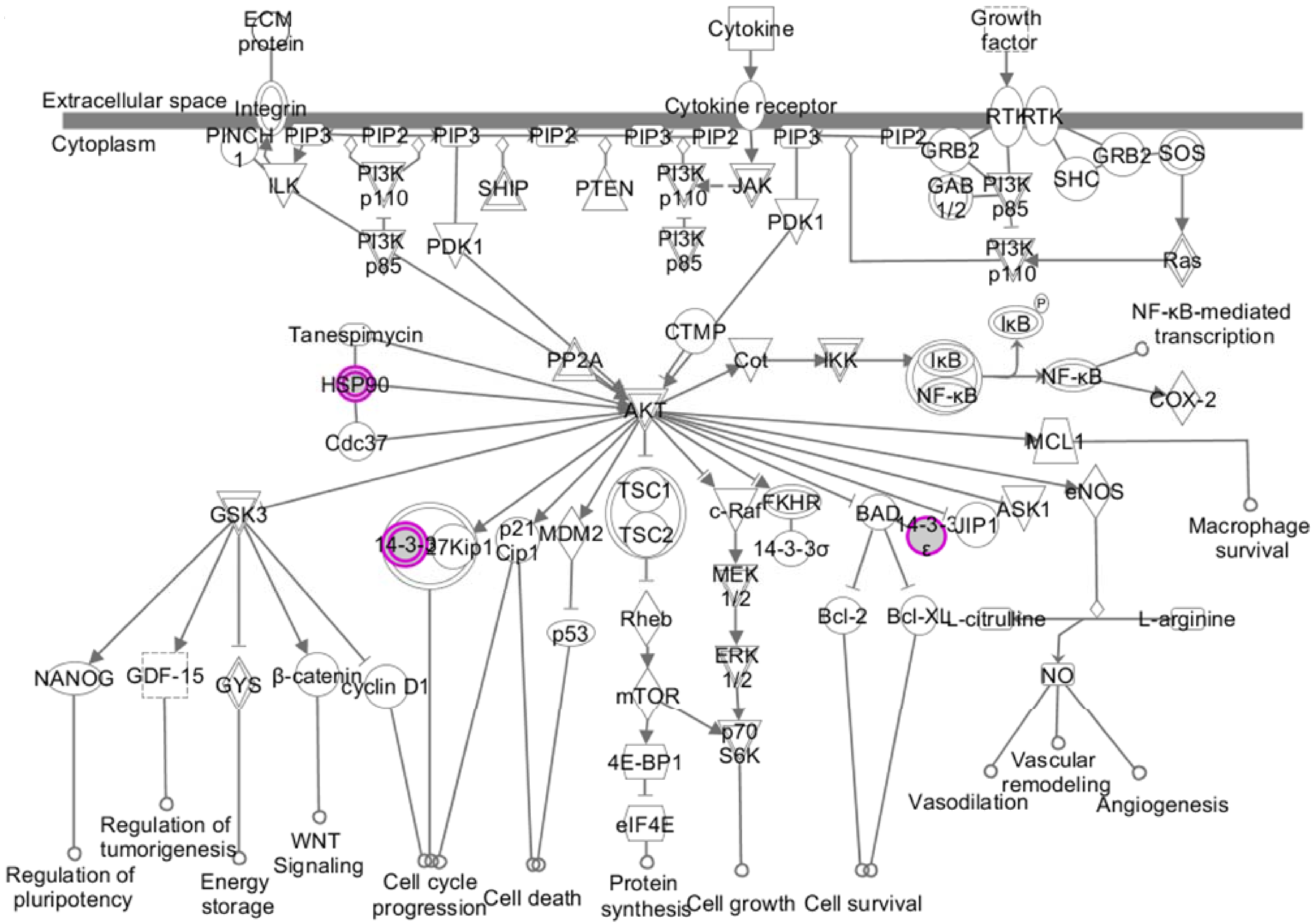

26. PPAR signaling

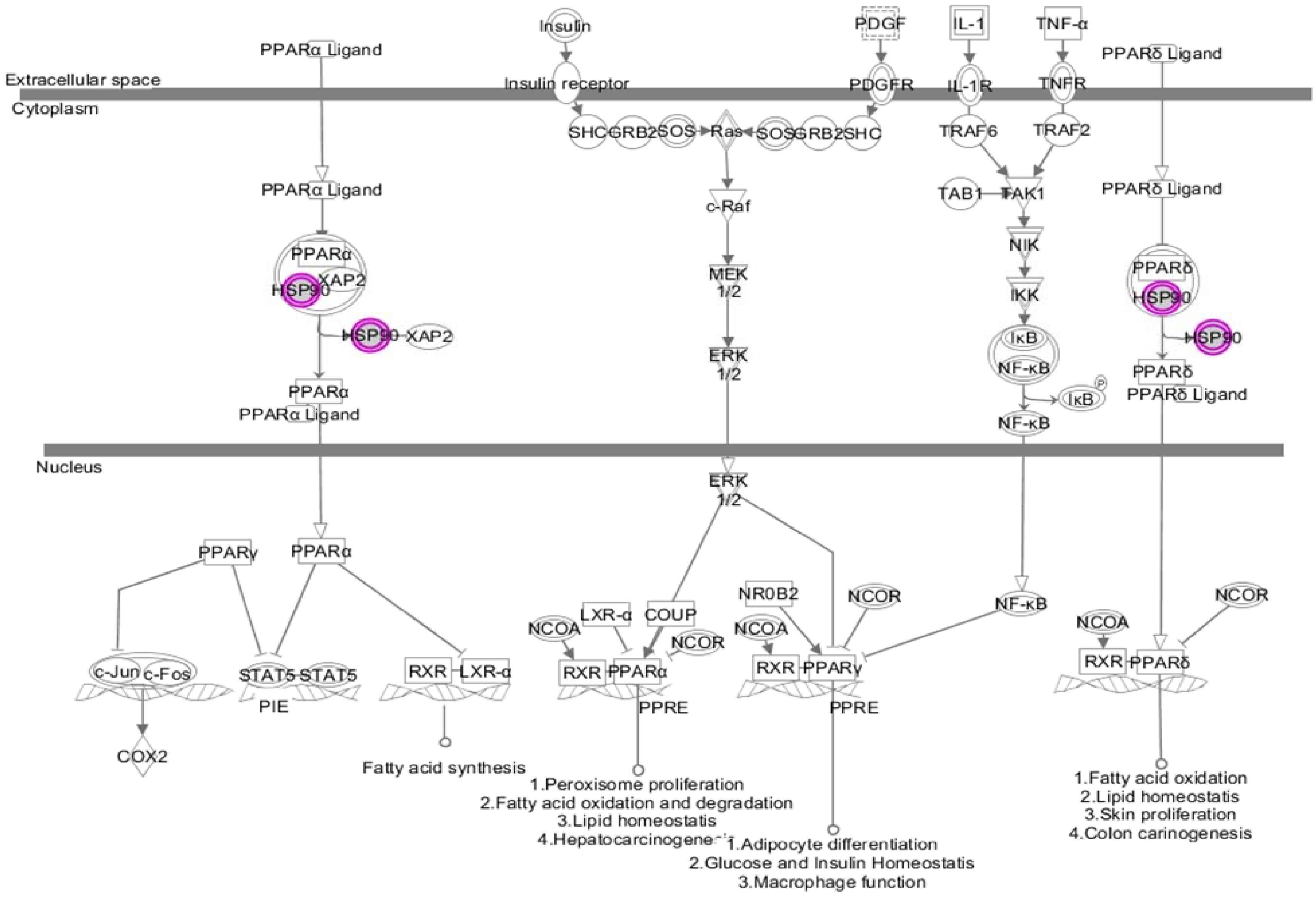

27. PPARa-RXRa activation

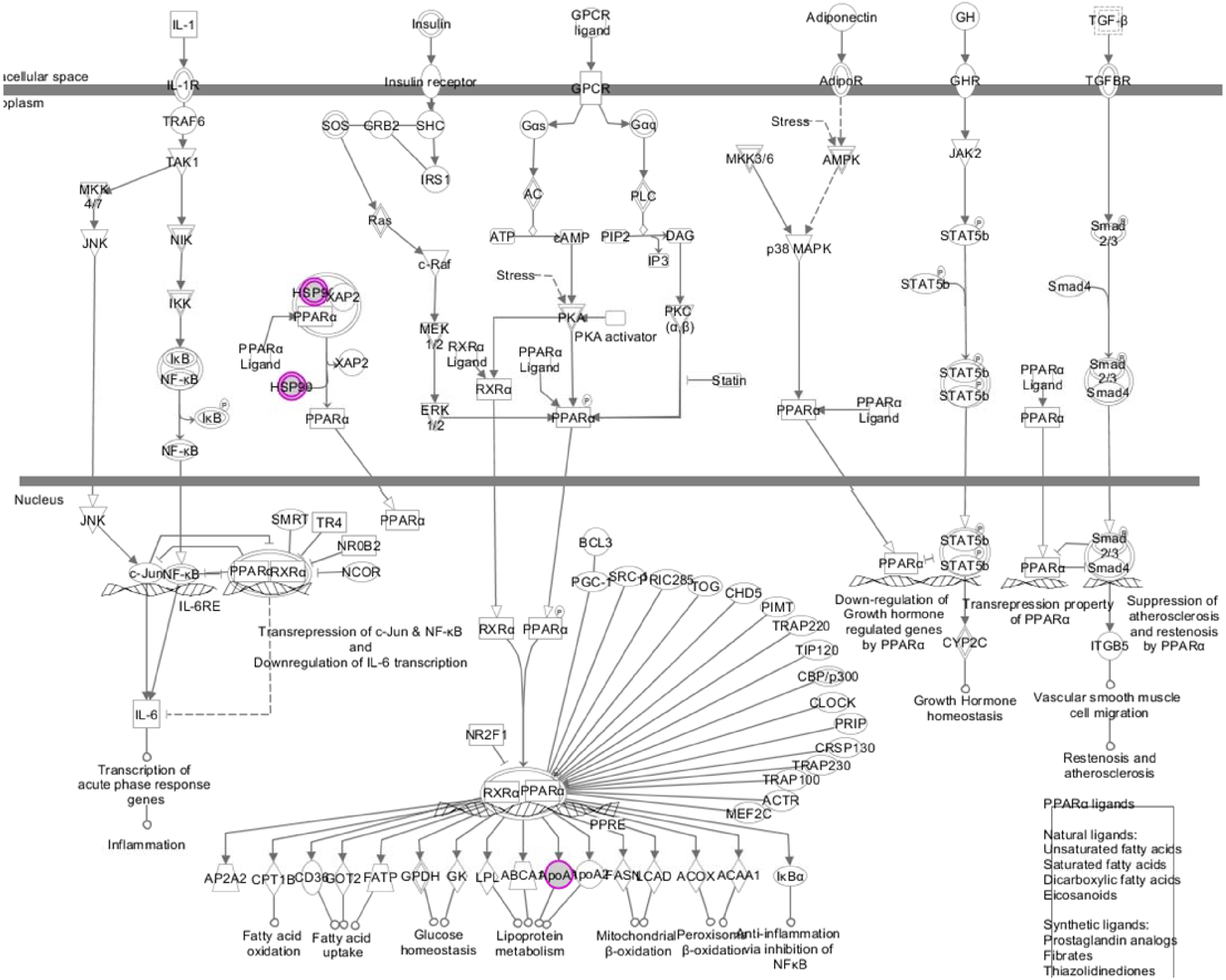

28. Prostate cancer signaling

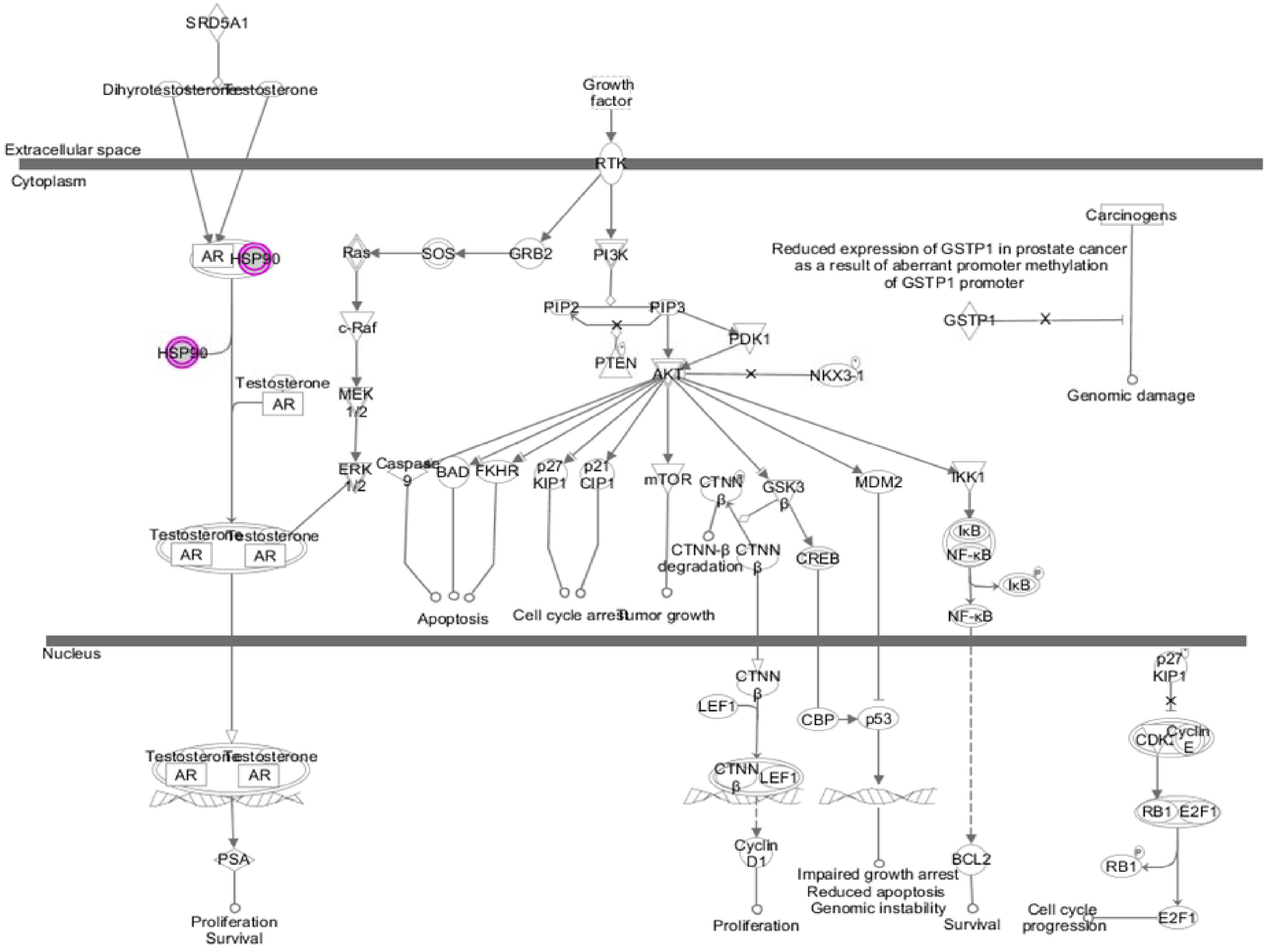

29. Protein ubiquitination pathway

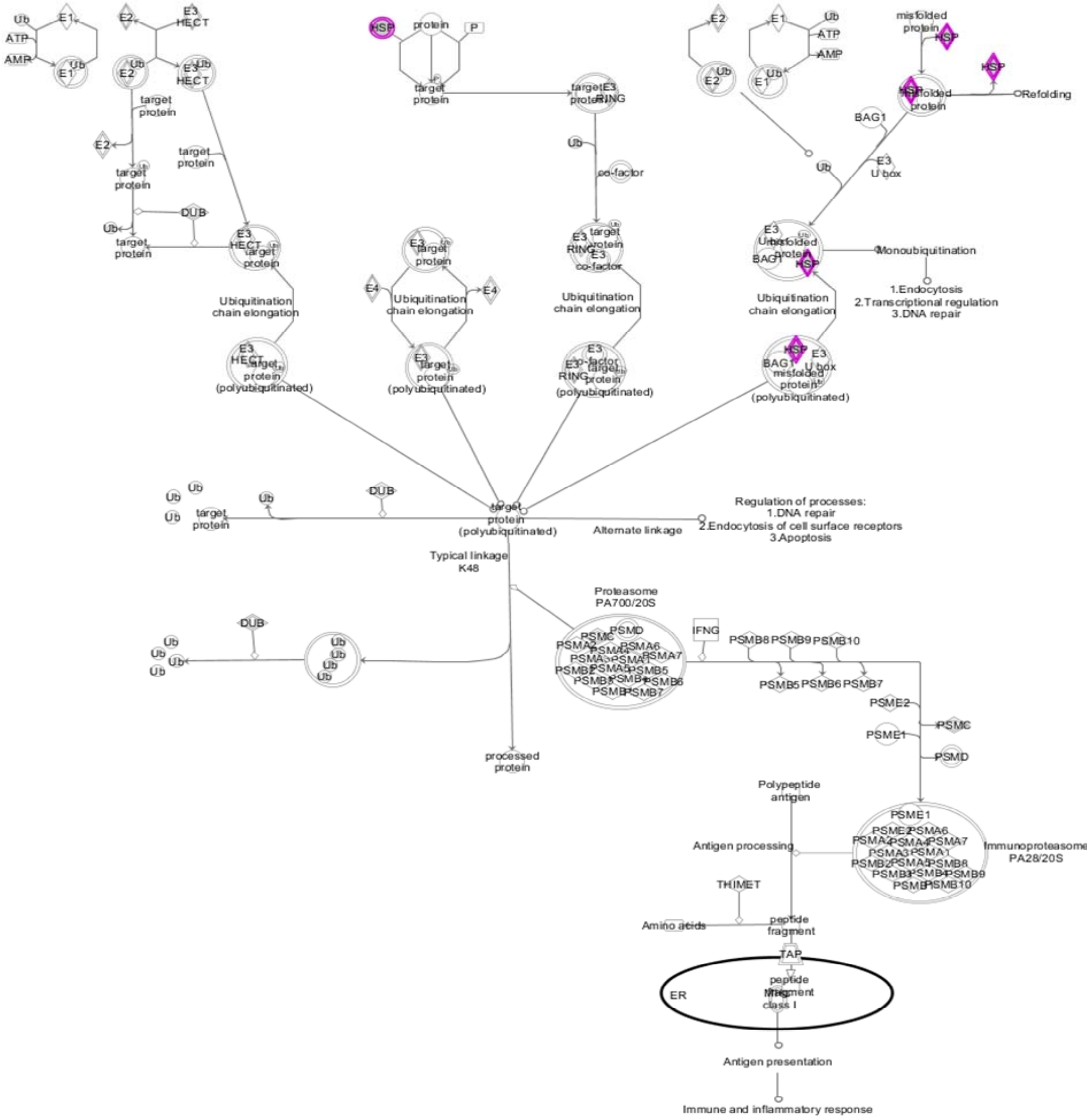

30. Pyruvate fermentation to lactate

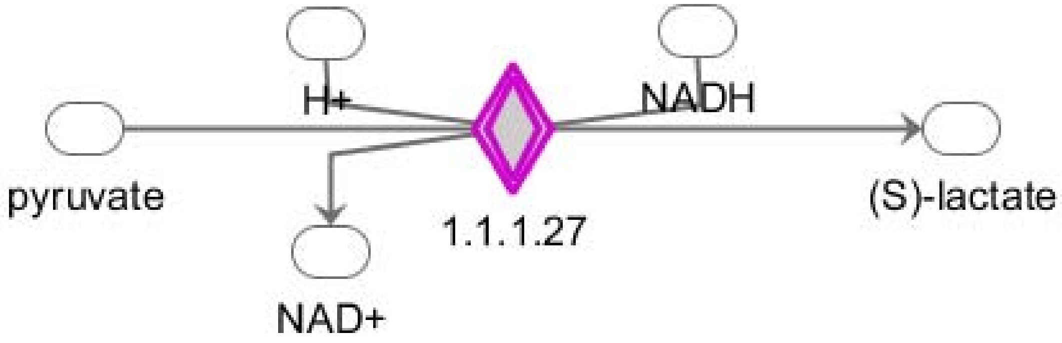

31. Remodeling of epithelial adherens junctions

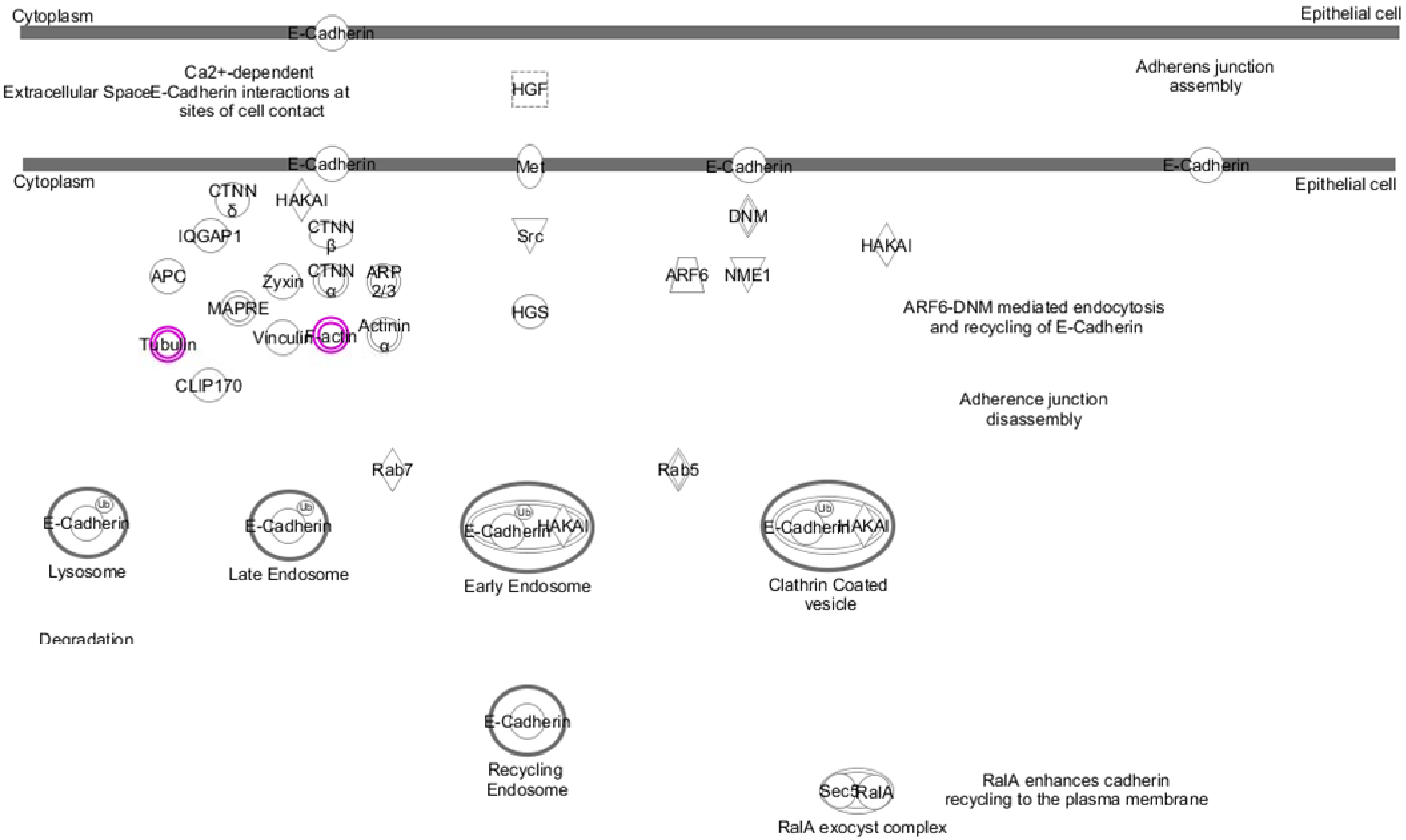

32. Sertoli cell-sertoli cell junction signaling

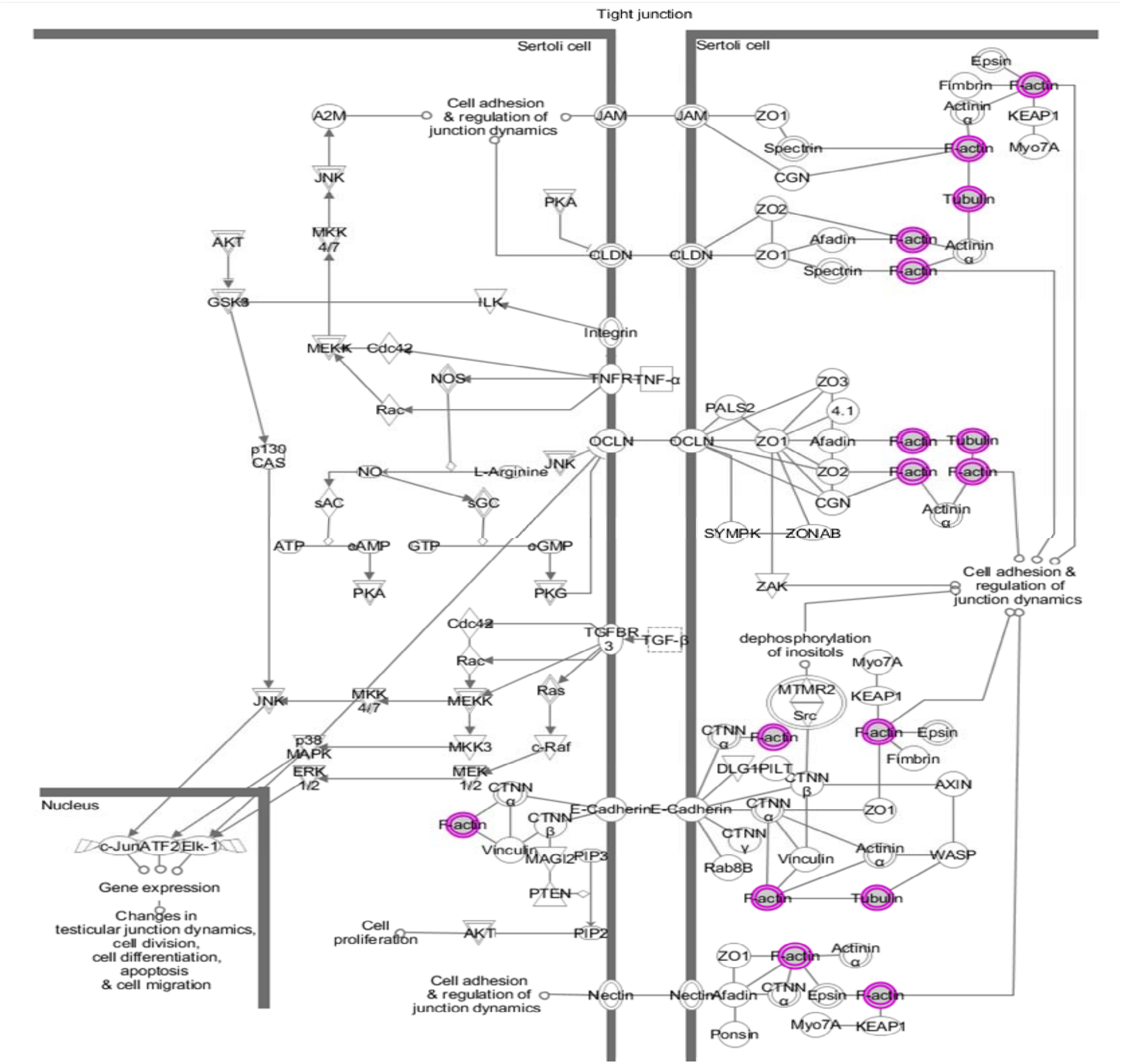

33. Signaling by Rho family GTPases

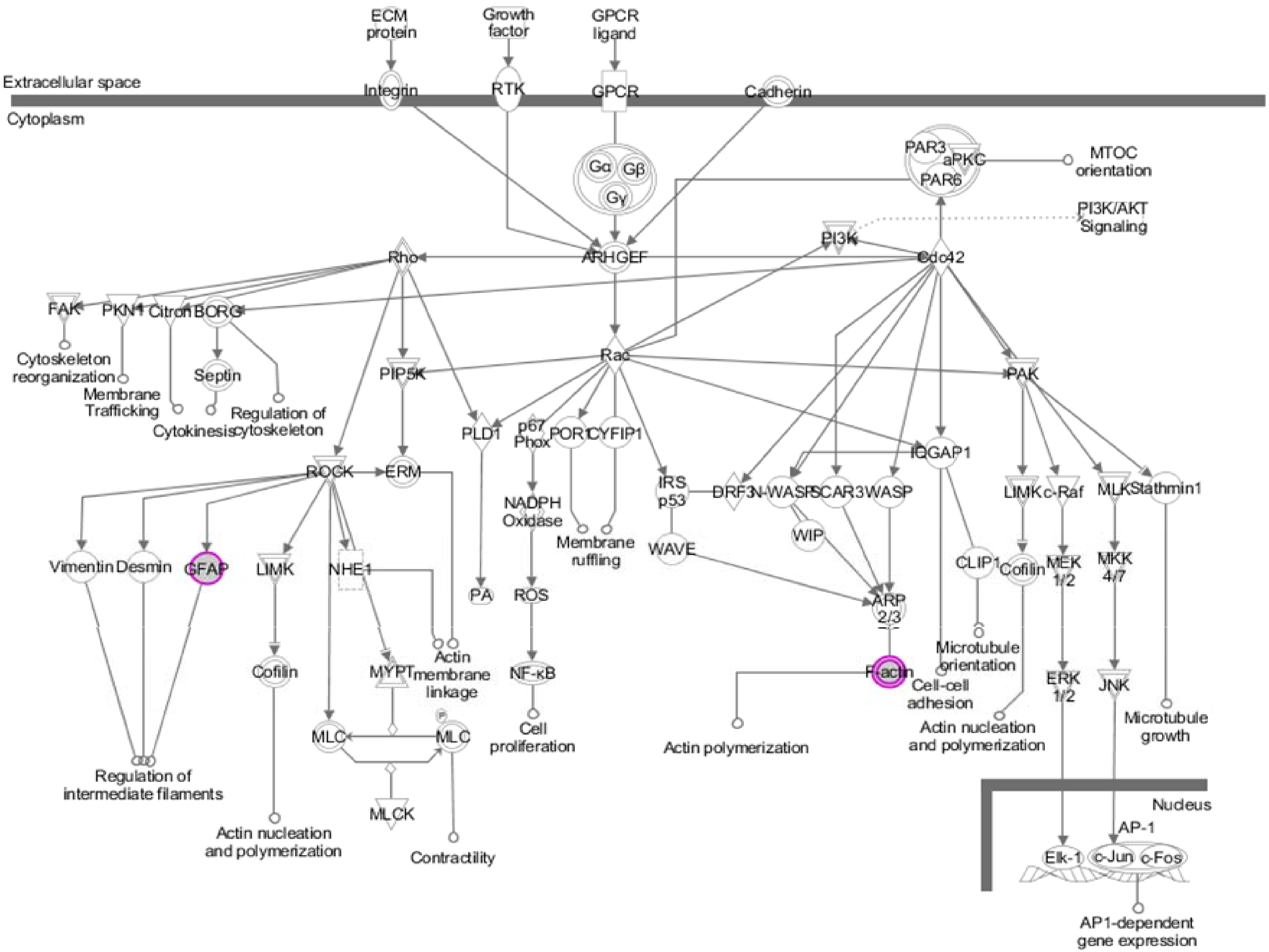

34. Telomerase signaling

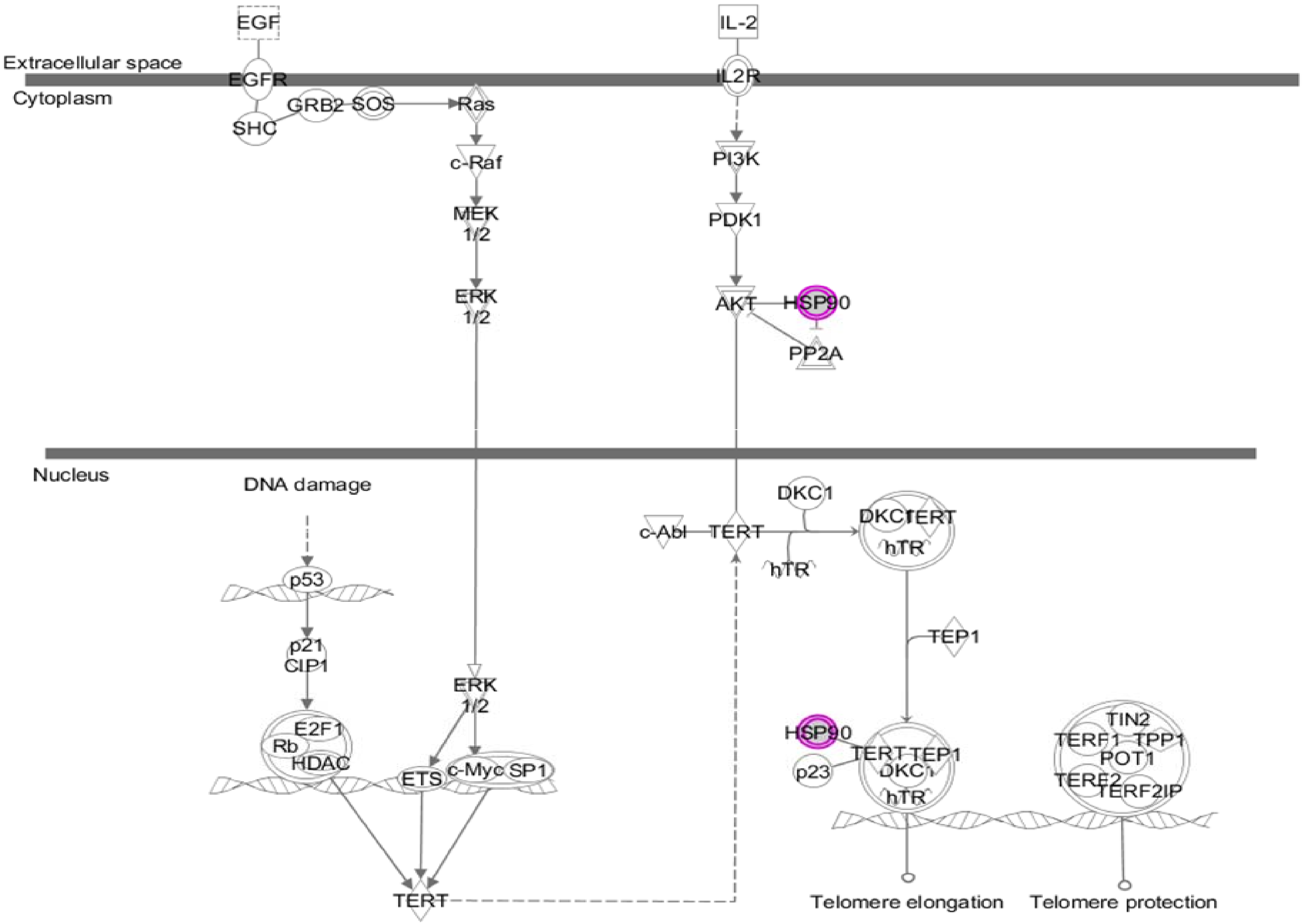

35. VEGF signaling

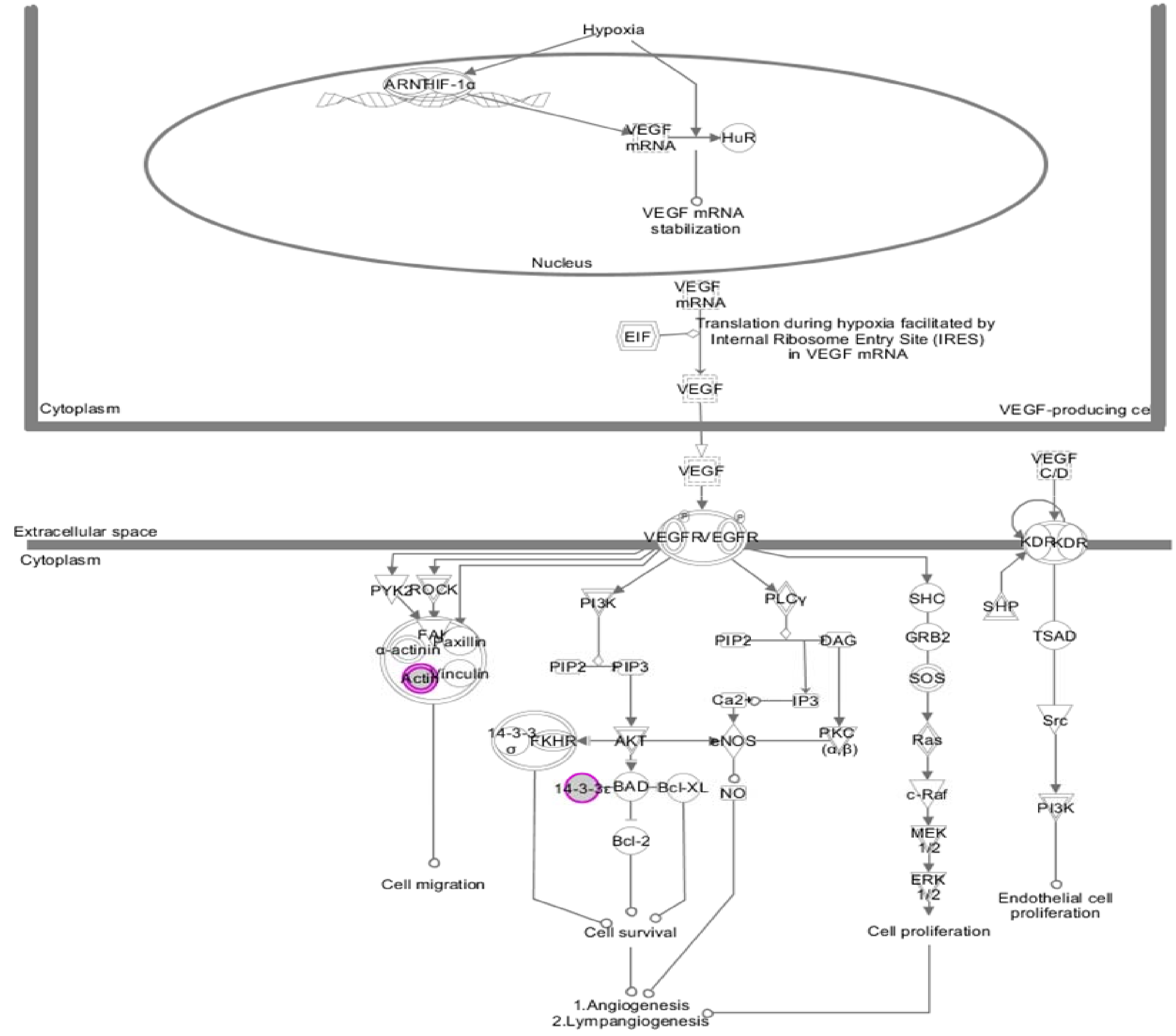

36. Xenobiotic metabolism signaling

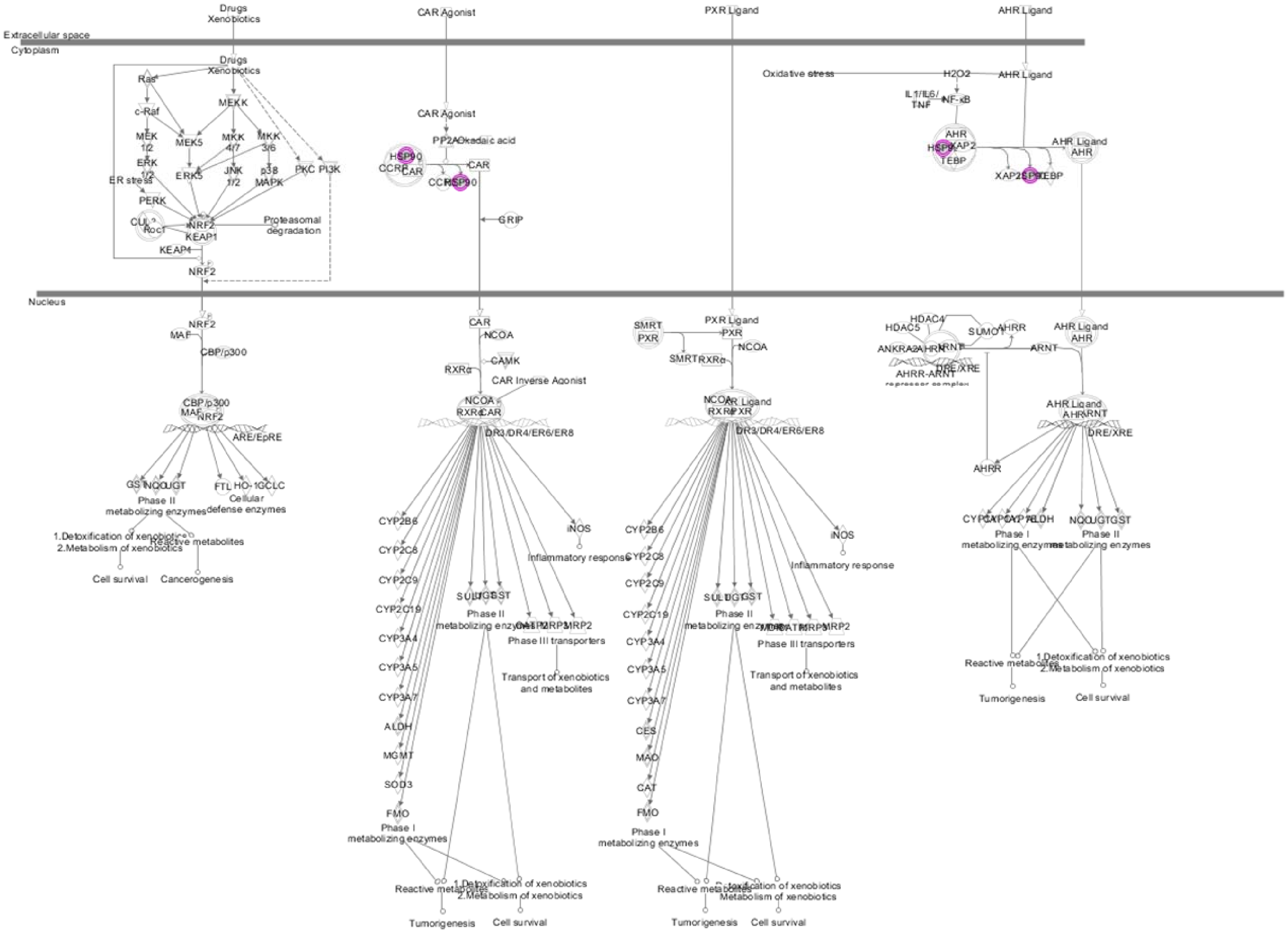

Supplement: Supplementary file 1 — Supplemental Table 1: Proteins without tyrosine phosphorylated motif, identified with 2DGE-based Western blotting and MS/MS Supplemental Table 2: Phosphotyrosine-containing proteins identified in a human glioblastoma tissue for IPA analysis Supplemental Table 3: Signaling pathway networks that involve phosphotyrosine-containing proteins identified from human glioblastoma Supplemental Figure 1: Significant canonical pathways involved phosphotyrosine-containing proteins in a glioblastoma tissue [file 134050.f1.pdf]
